# Supplementary material for: Gene therapy for epilepsy targeting neuropeptide Y and its Y2 receptor to dentate gyrus granule cells
Source: EMBO Rep. 2024 Sep 9;25(10):20. doi: 10.1038/s44319-024-00244-0 (PMC11467199; doi:10.1038/s44319-024-00244-0)
Supplement: Supplementary file 2 — Source data Fig. 2 [file 44319_2024_244_MOESM2_ESM.zip › Figure 2/2D/Figure 2D.pptx]

## Slide 1
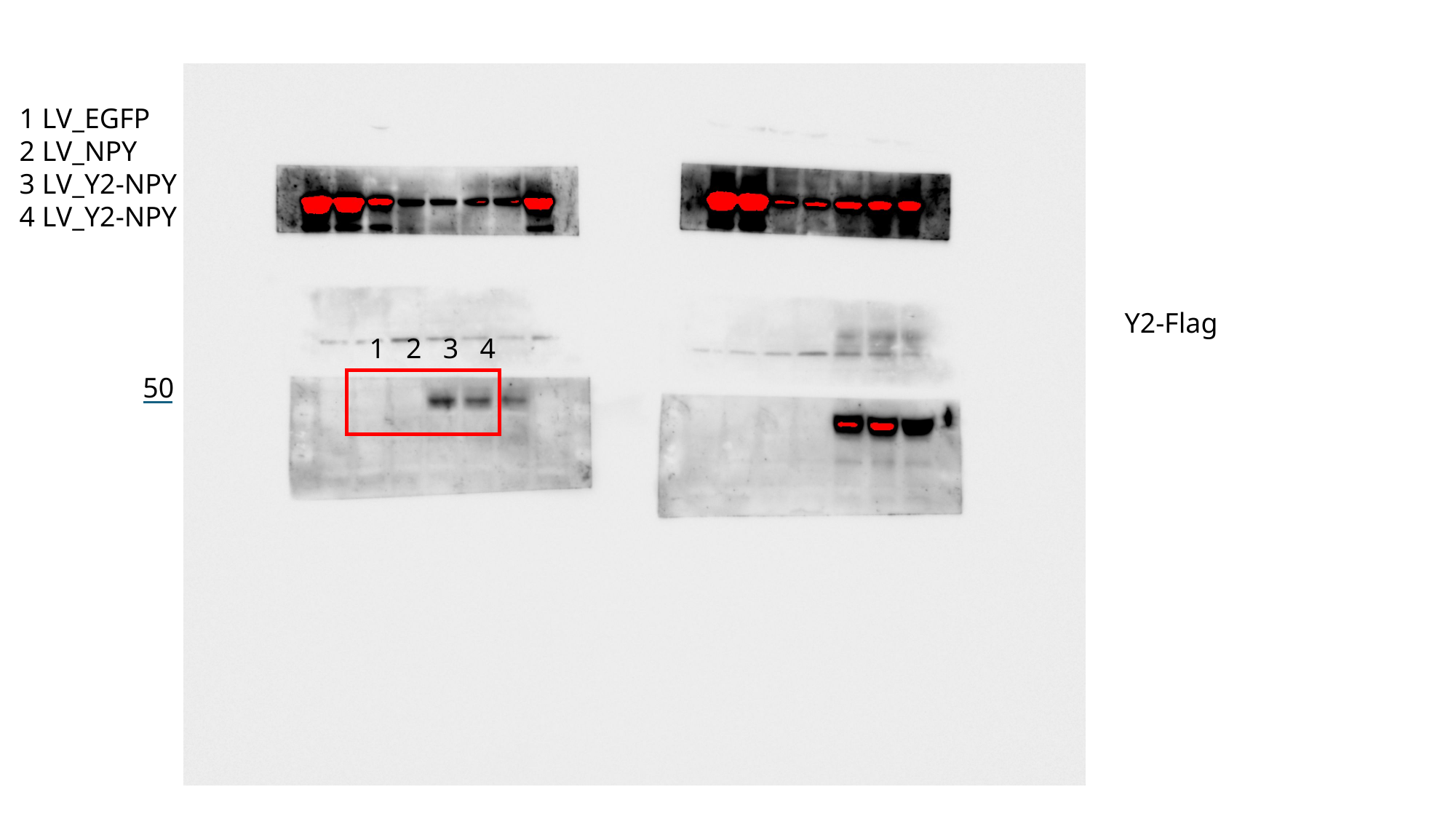

1 LV_EGFP
2 LV_NPY
3 LV_Y2-NPY
4 LV_Y2-NPY
Y2-Flag
1 2 3 4
50

## Slide 2
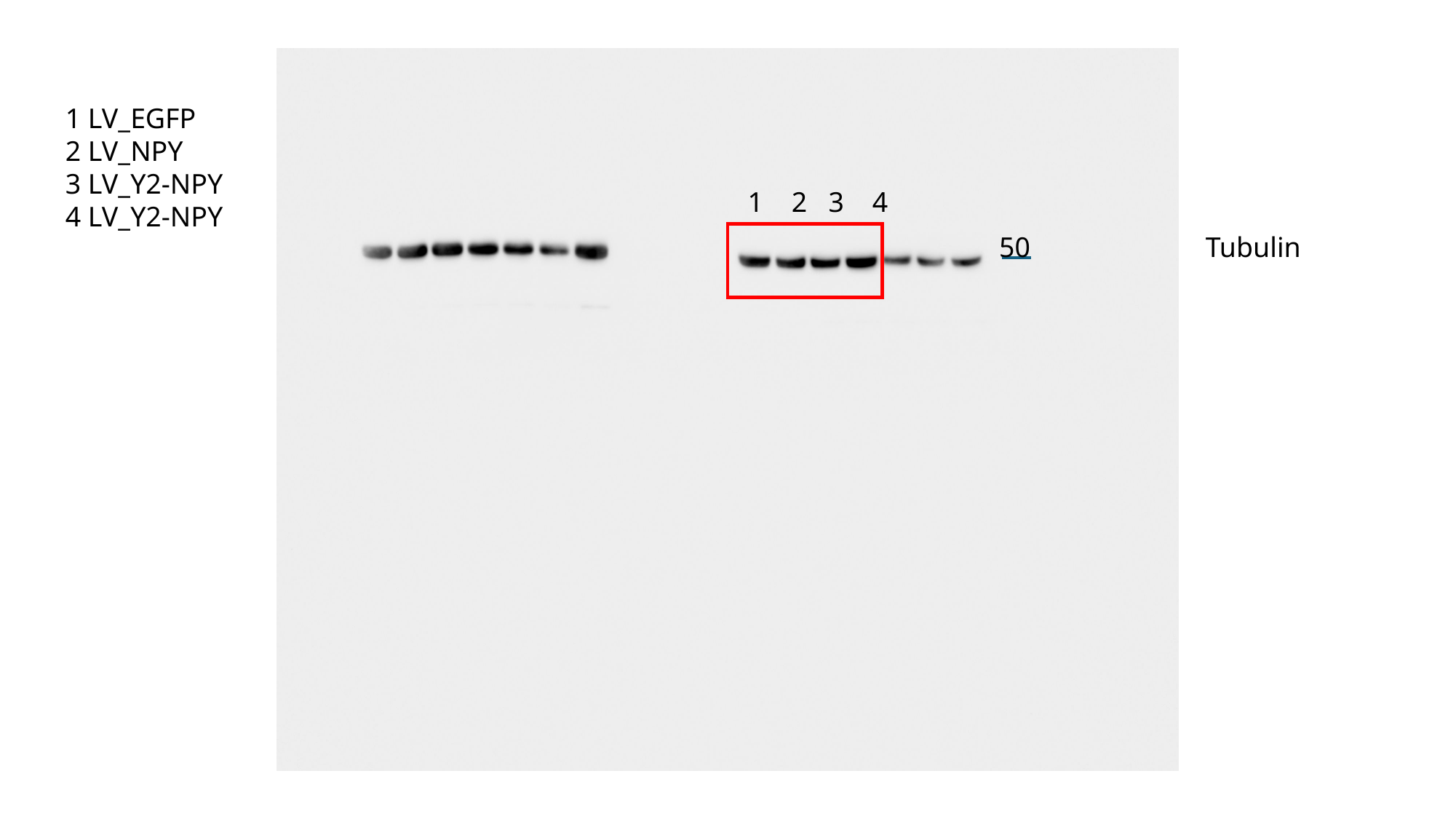

1 LV_EGFP
2 LV_NPY
3 LV_Y2-NPY
4 LV_Y2-NPY
1 2 3 4
50
Tubulin

## Slide 3
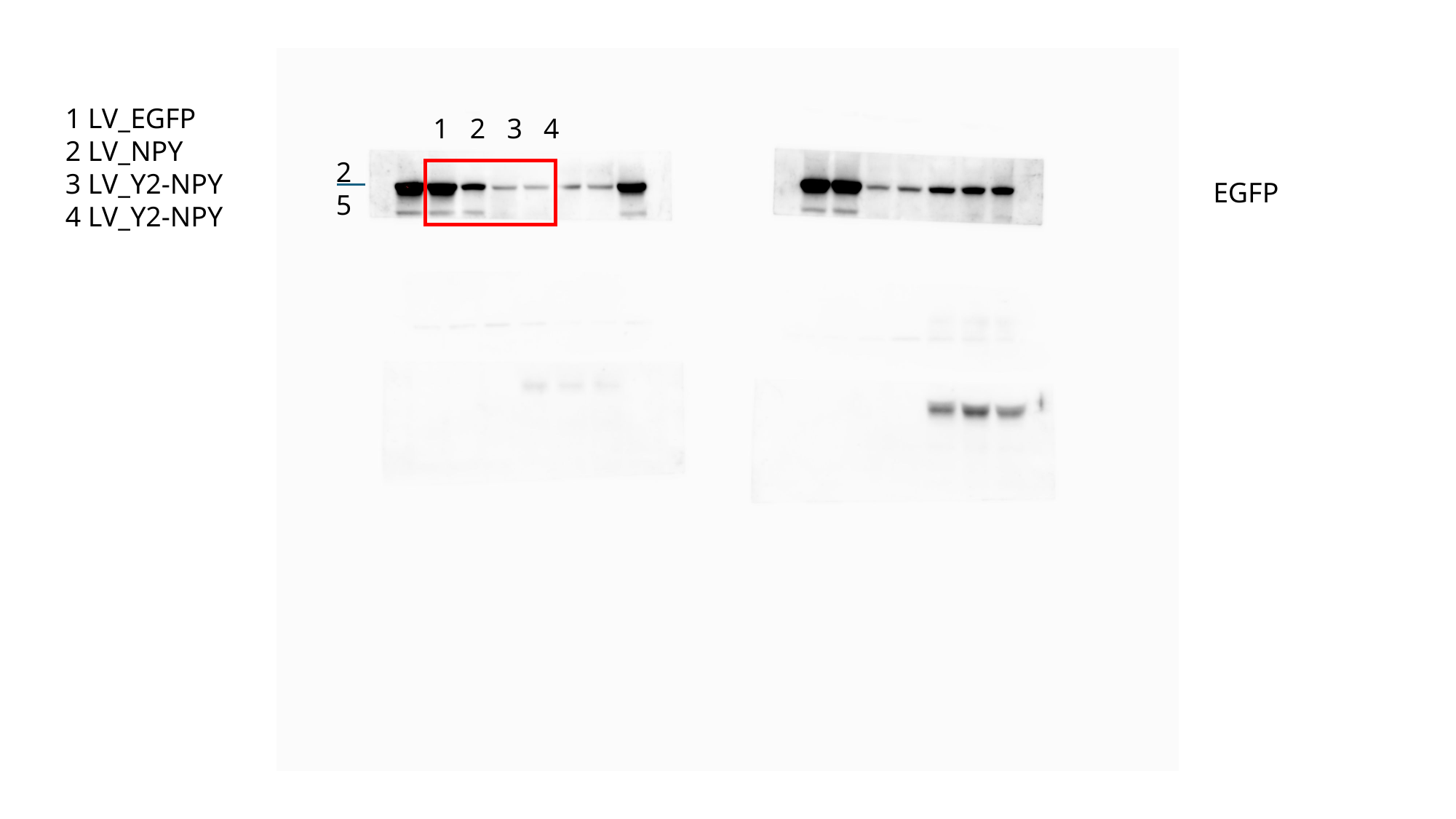

1 LV_EGFP
2 LV_NPY
3 LV_Y2-NPY
4 LV_Y2-NPY
1 2 3 4
25
EGFP

## Slide 4
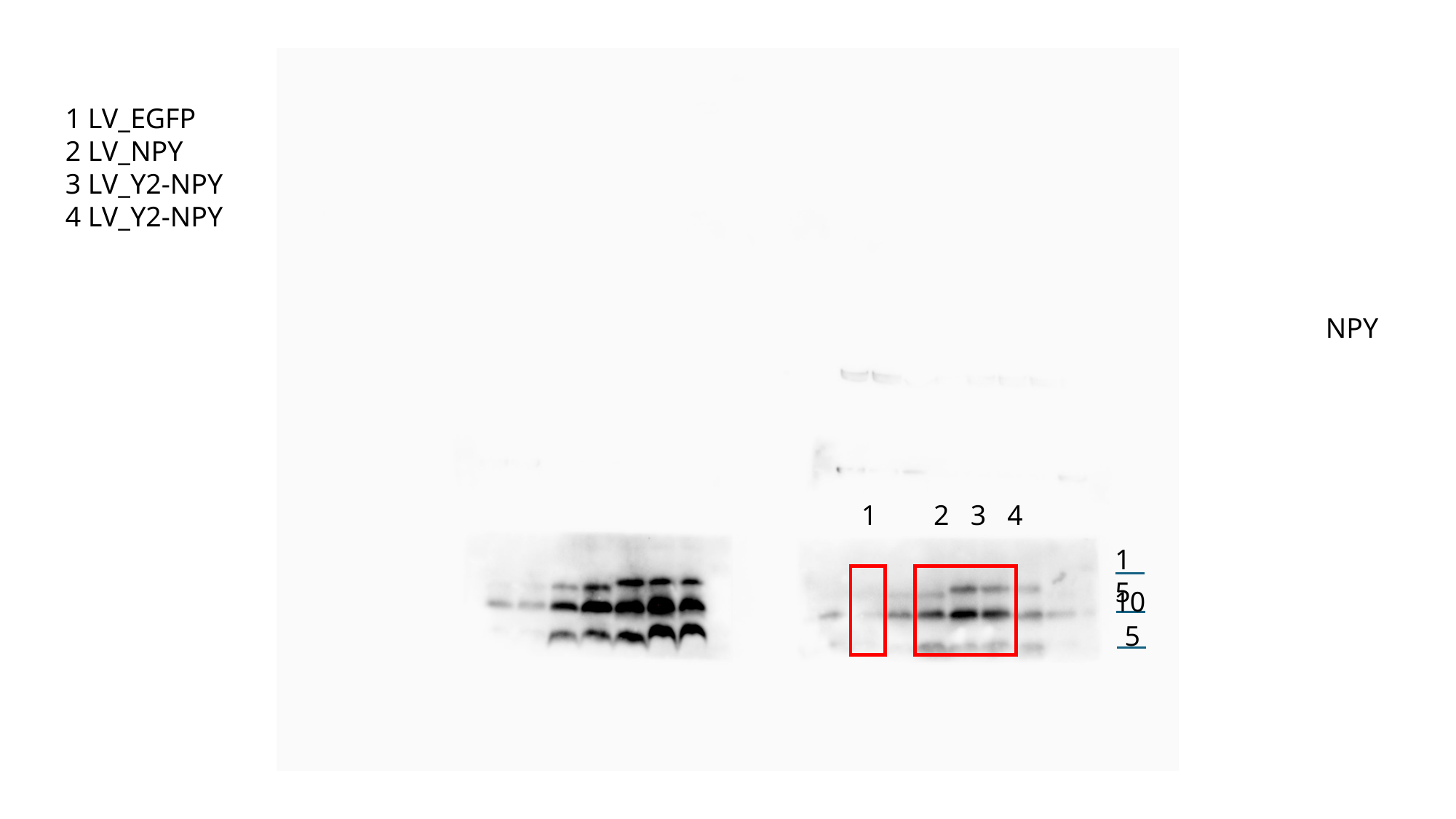

1 LV_EGFP
2 LV_NPY
3 LV_Y2-NPY
4 LV_Y2-NPY
NPY
1 2 3 4
15
10
5
