# Supplementary figures and images for: Gene therapy for epilepsy targeting neuropeptide Y and its Y2 receptor to dentate gyrus granule cells
Source: EMBO Rep. 2024 Sep 9;25(10):20. doi: 10.1038/s44319-024-00244-0 (PMC11467199; doi:10.1038/s44319-024-00244-0)

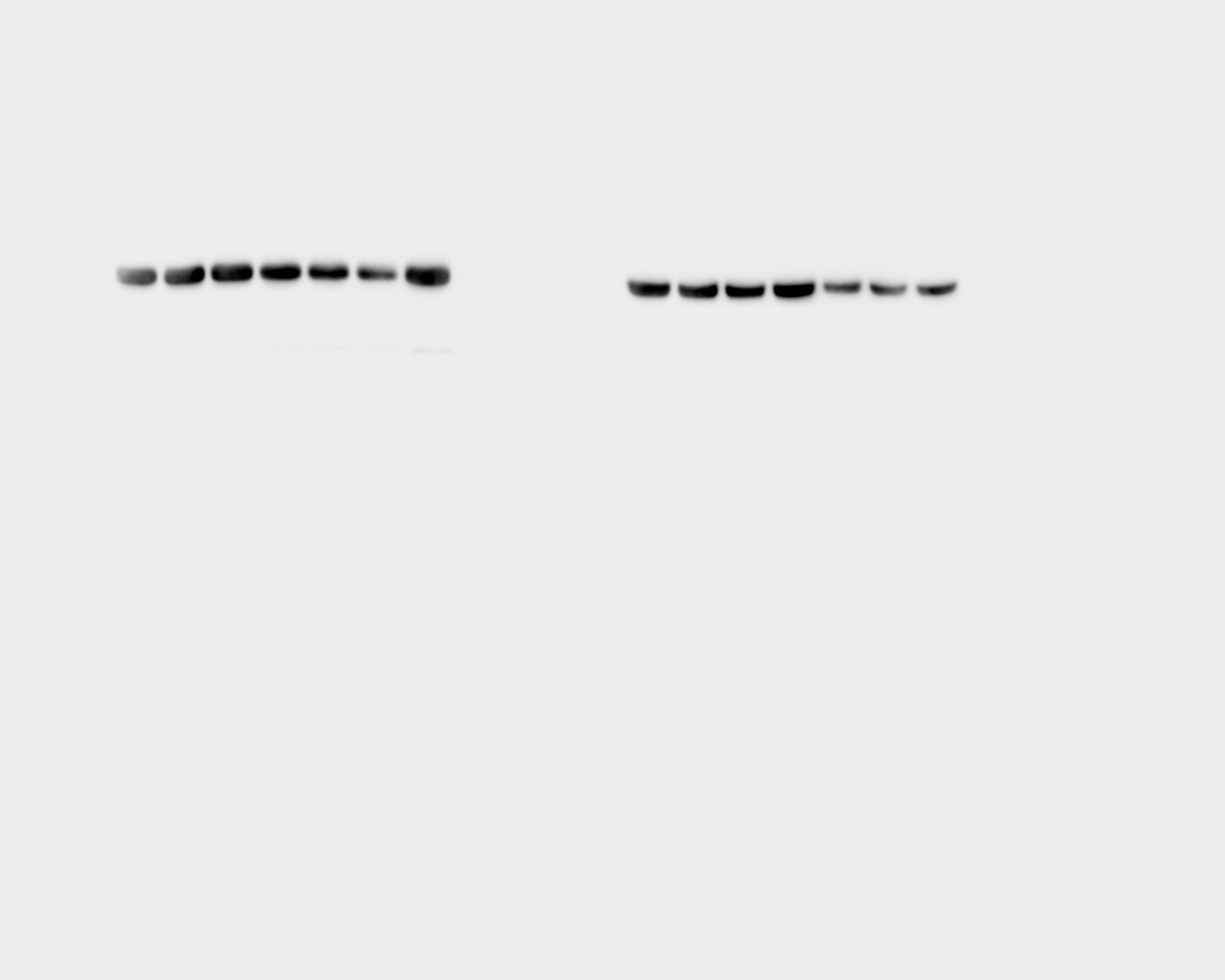

Supplement: Supplementary file 2 — Source data Fig. 2 [file 44319_2024_244_MOESM2_ESM.zip › Figure 2/2D/Tubulin.tif]

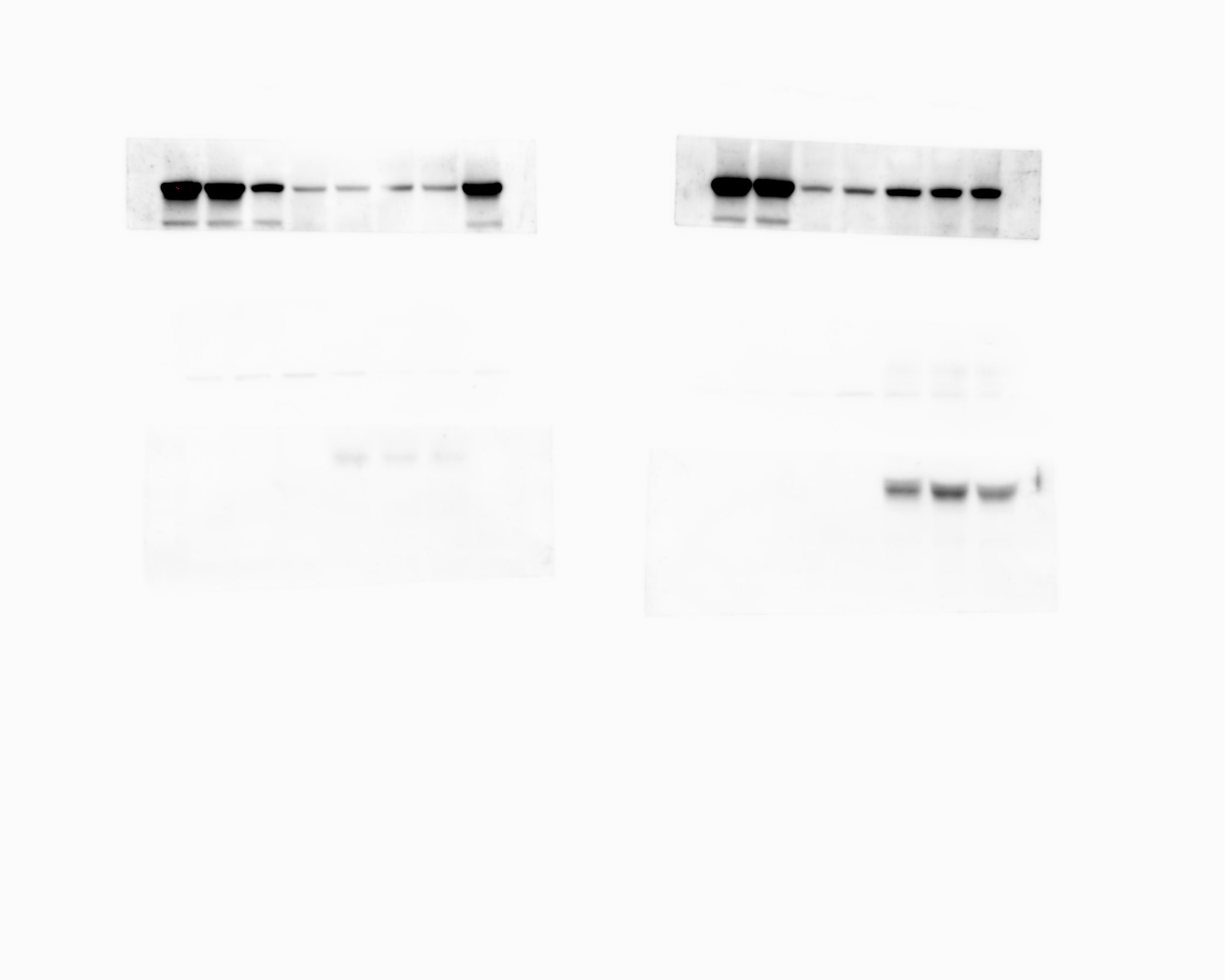

Supplement: Supplementary file 2 — Source data Fig. 2 [file 44319_2024_244_MOESM2_ESM.zip › Figure 2/2D/EGFP.tif]

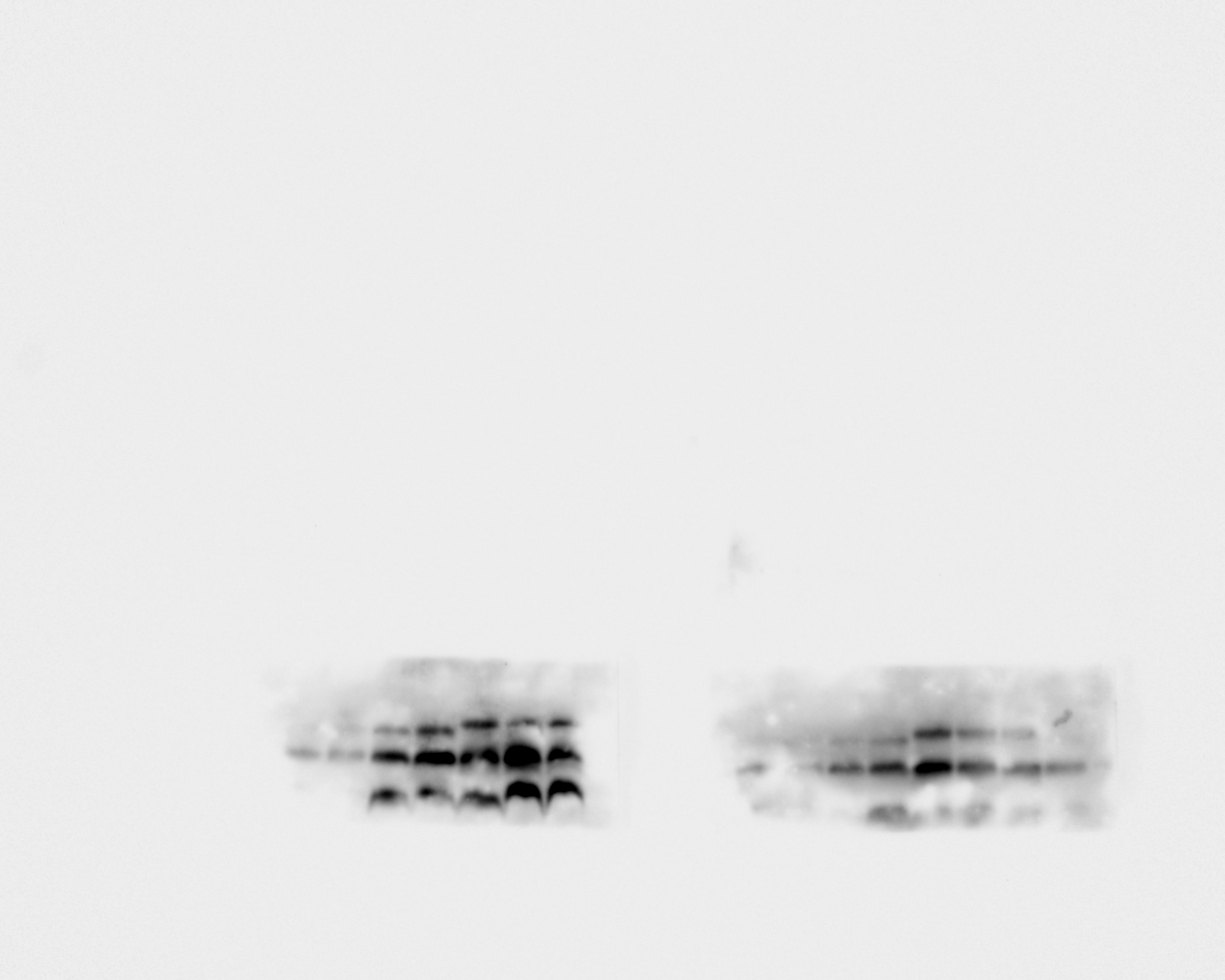

Supplement: Supplementary file 2 — Source data Fig. 2 [file 44319_2024_244_MOESM2_ESM.zip › Figure 2/2D/NPY.tif]

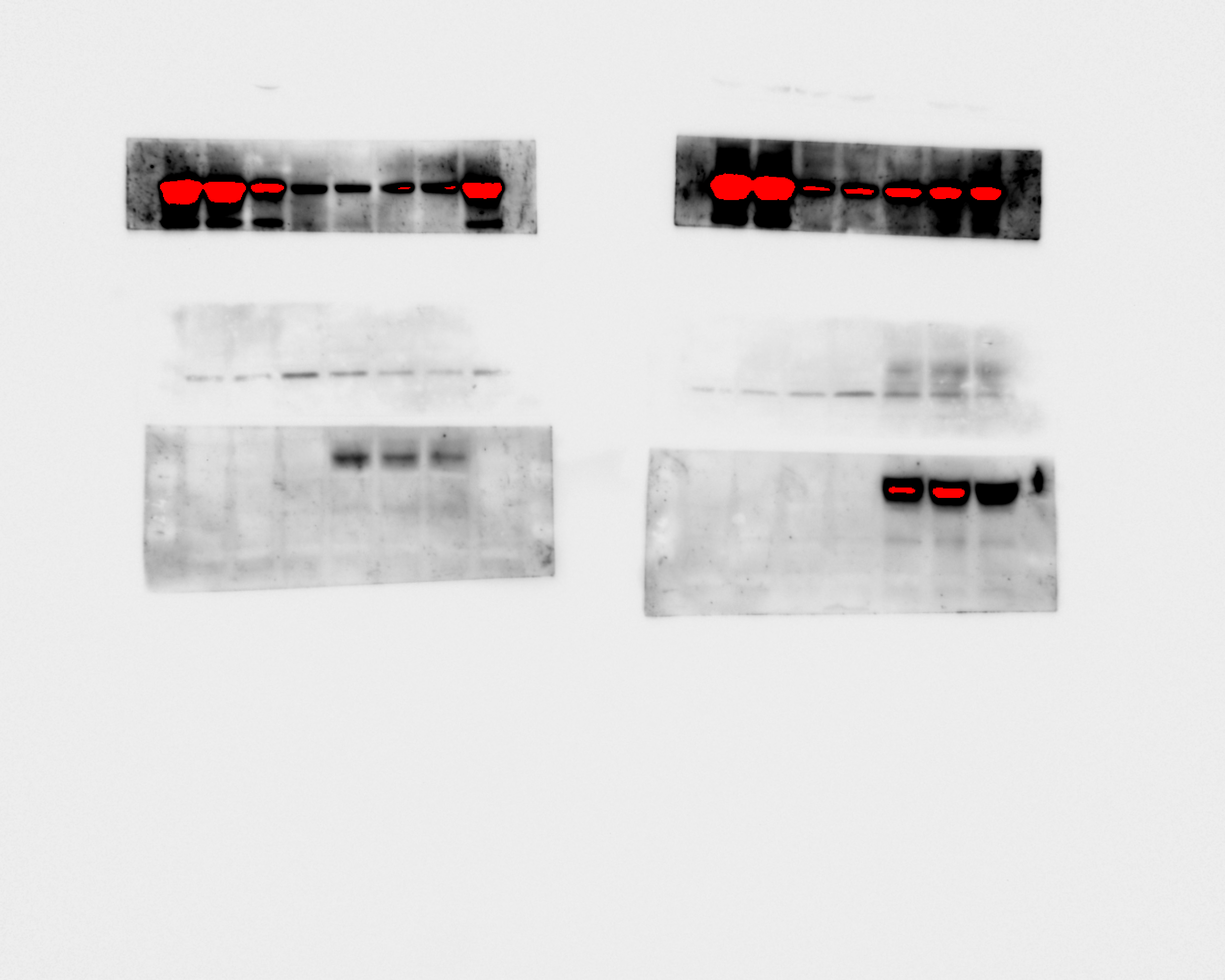

Supplement: Supplementary file 2 — Source data Fig. 2 [file 44319_2024_244_MOESM2_ESM.zip › Figure 2/2D/Y2.tif]

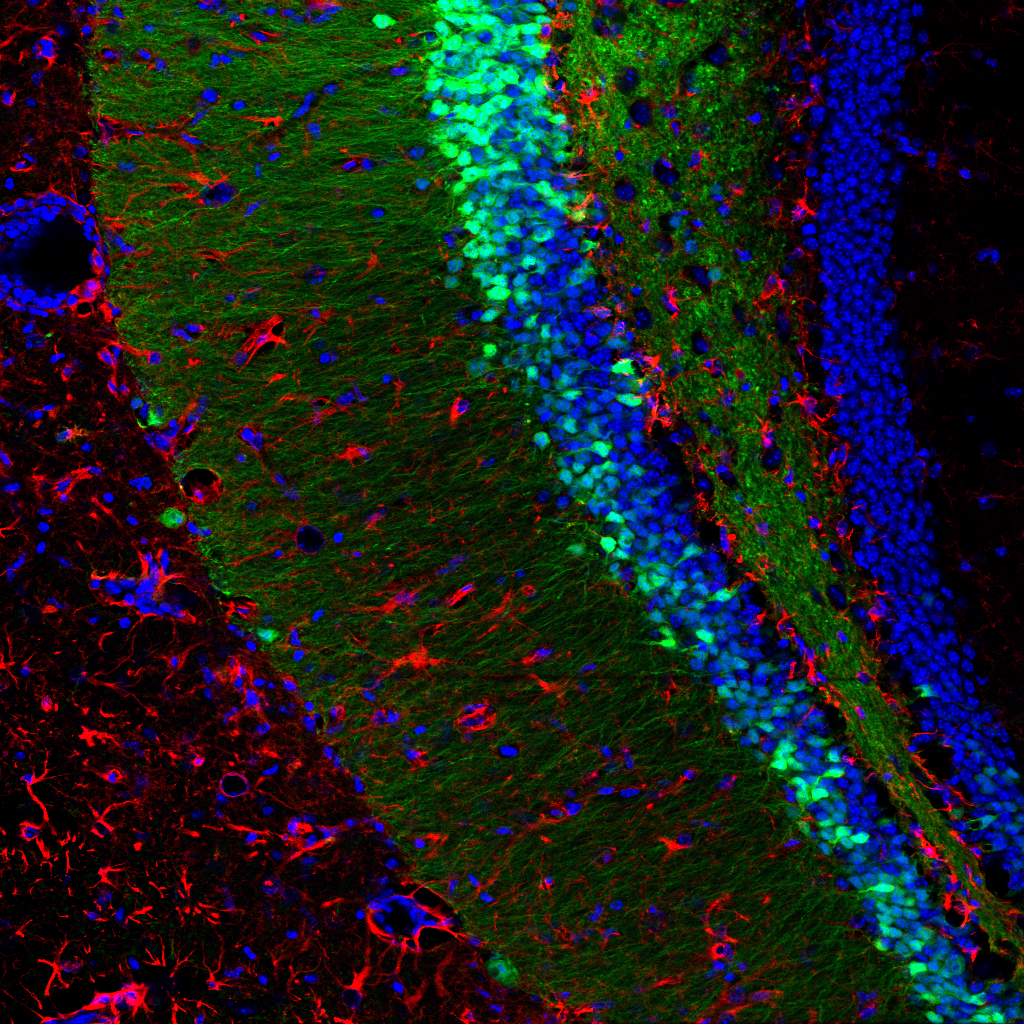

Supplement: Supplementary file 3 — Source data Fig. 3 [file 44319_2024_244_MOESM3_ESM.zip › Figure 3/3E/3E-E'''.tif]

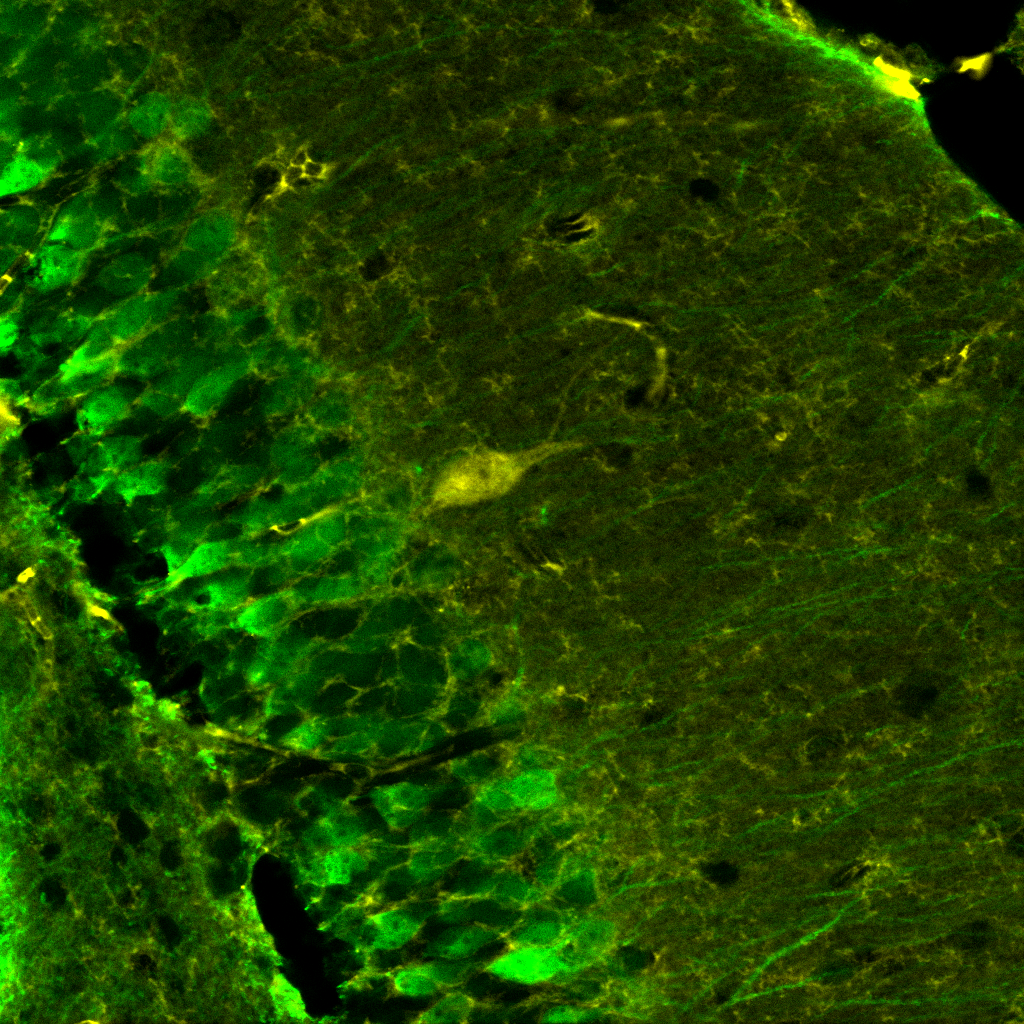

Supplement: Supplementary file 4 — Source data Fig. 4 [file 44319_2024_244_MOESM4_ESM.zip › Figure 4/4B/4B-B''.tif]

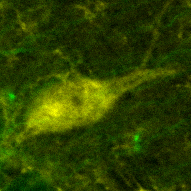

Supplement: Supplementary file 4 — Source data Fig. 4 [file 44319_2024_244_MOESM4_ESM.zip › Figure 4/4B/4B-B'' inset.tif]

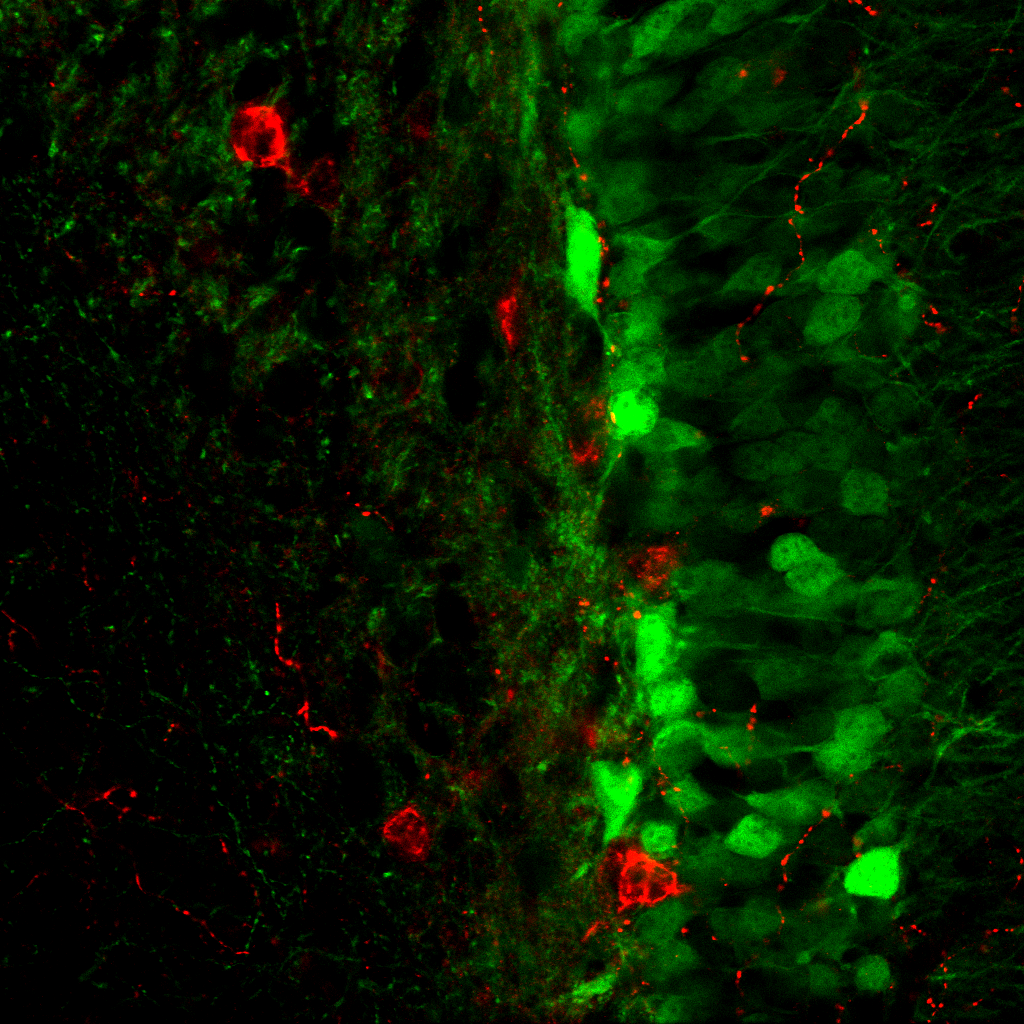

Supplement: Supplementary file 4 — Source data Fig. 4 [file 44319_2024_244_MOESM4_ESM.zip › Figure 4/4C/4C-C''.tif]

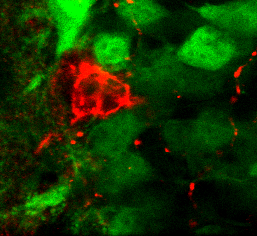

Supplement: Supplementary file 4 — Source data Fig. 4 [file 44319_2024_244_MOESM4_ESM.zip › Figure 4/4C/4C-C'' inset.tif]

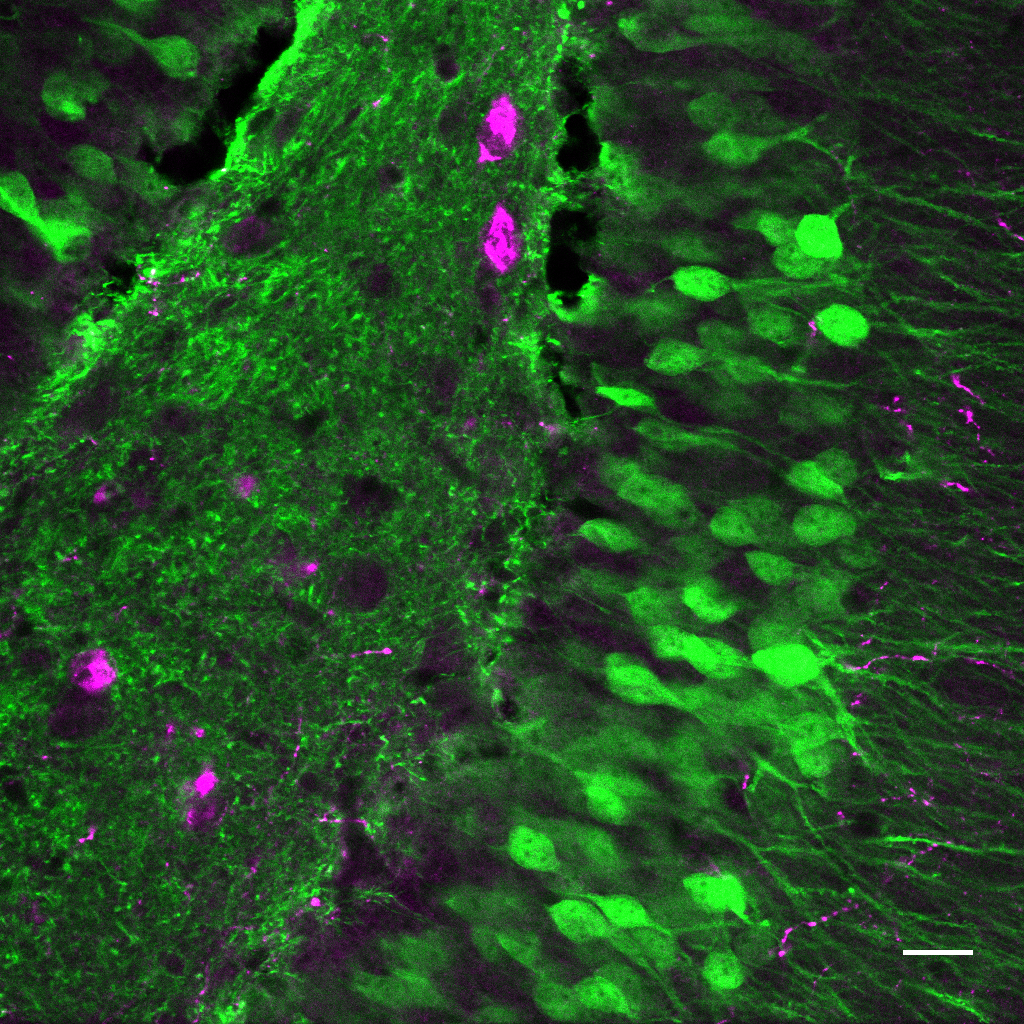

Supplement: Supplementary file 4 — Source data Fig. 4 [file 44319_2024_244_MOESM4_ESM.zip › Figure 4/4D/4D-D''.tif]

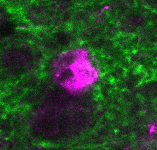

Supplement: Supplementary file 4 — Source data Fig. 4 [file 44319_2024_244_MOESM4_ESM.zip › Figure 4/4D/4D-D'' inset.tif]

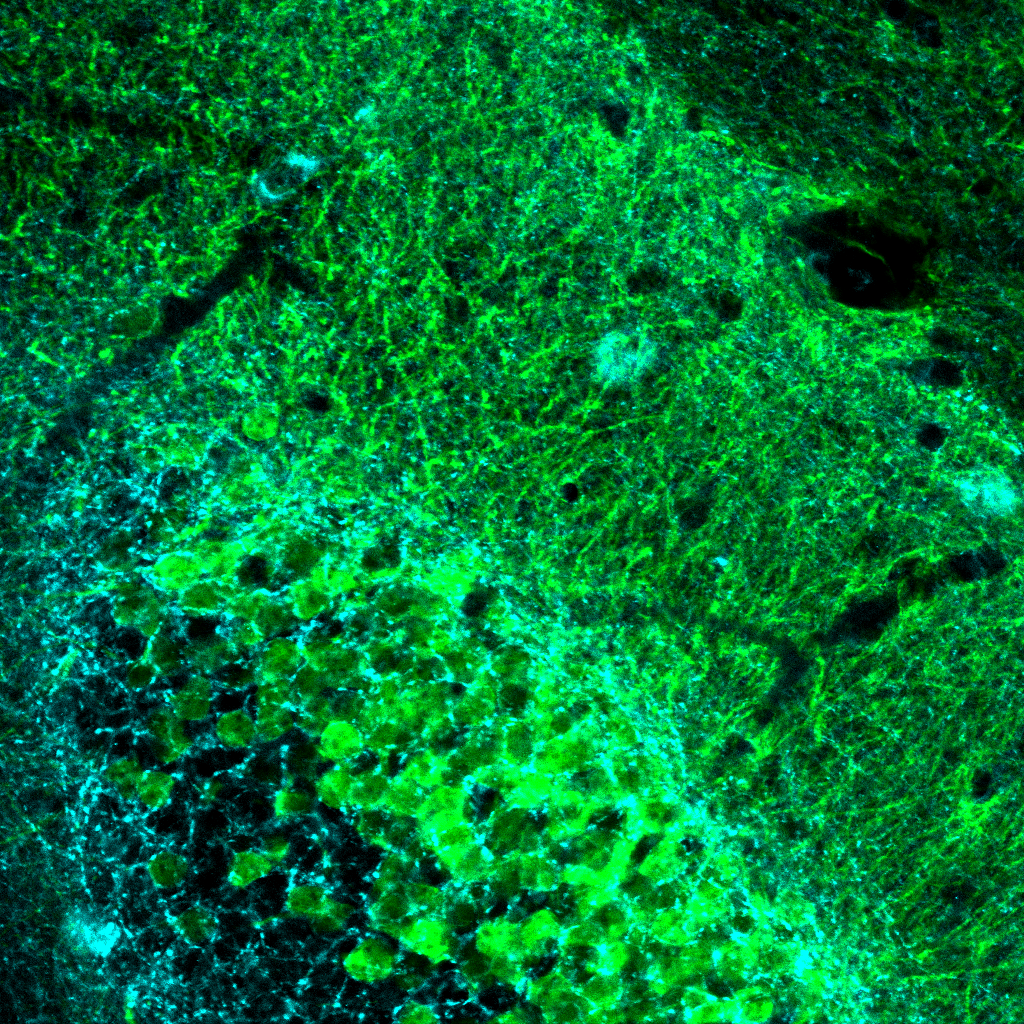

Supplement: Supplementary file 4 — Source data Fig. 4 [file 44319_2024_244_MOESM4_ESM.zip › Figure 4/4A/4A-A''.tif]

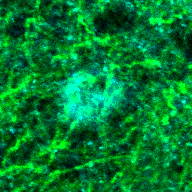

Supplement: Supplementary file 4 — Source data Fig. 4 [file 44319_2024_244_MOESM4_ESM.zip › Figure 4/4A/4A-A'' inset.tif]

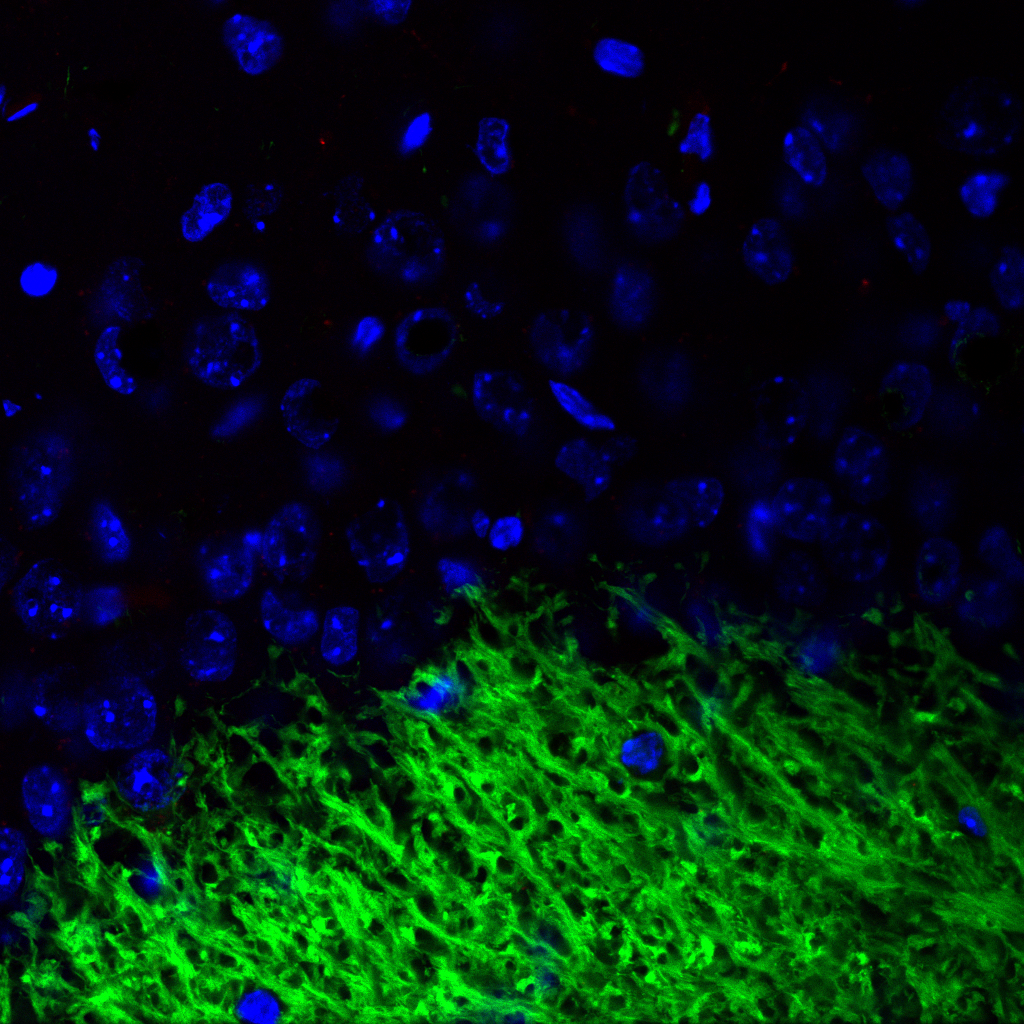

Supplement: Supplementary file 5 — Source data Fig. 5 [file 44319_2024_244_MOESM5_ESM.zip › Figure 5/5A/5A'''.tif]

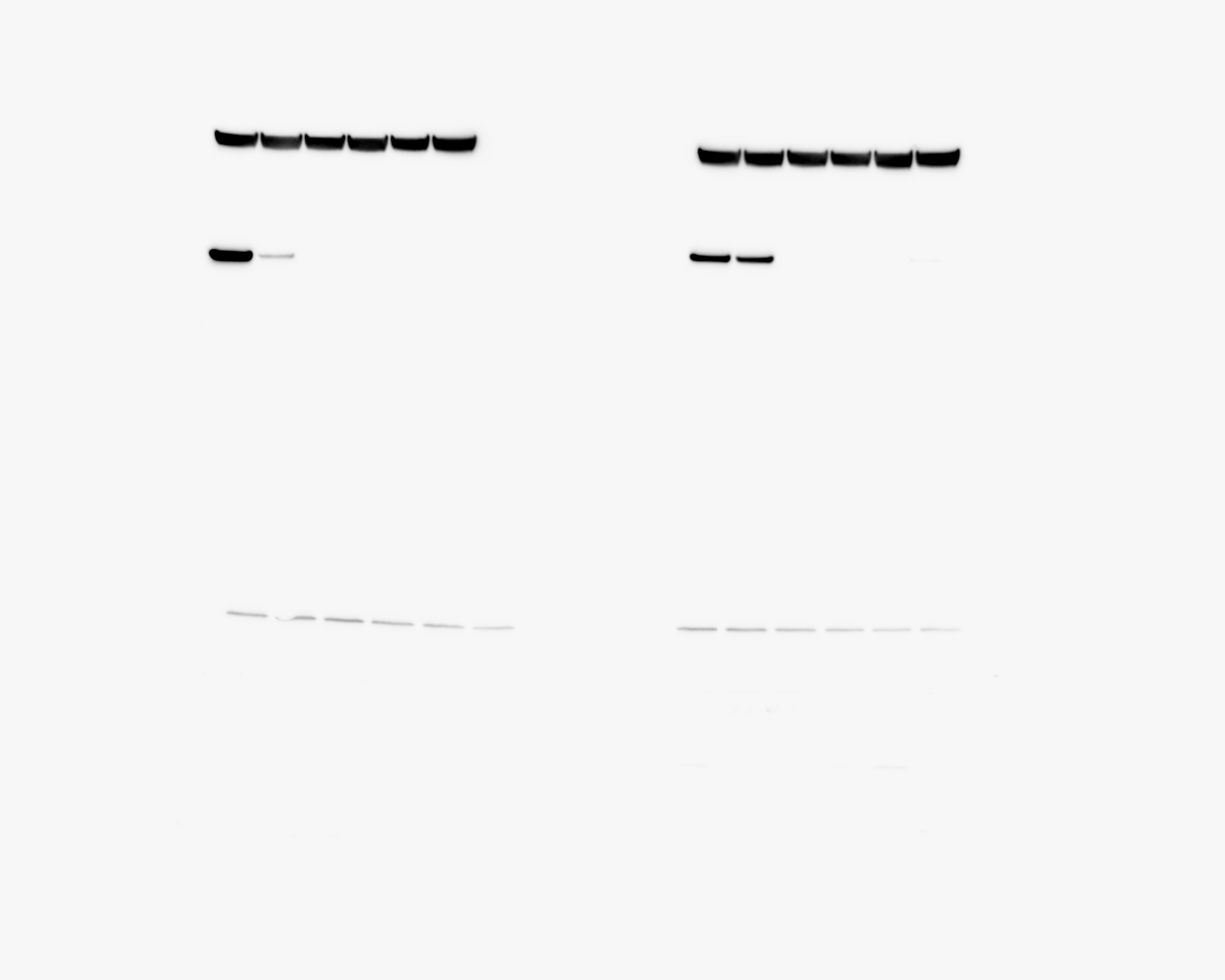

Supplement: Supplementary file 5 — Source data Fig. 5 [file 44319_2024_244_MOESM5_ESM.zip › Figure 5/5F/Tubulin.tif]

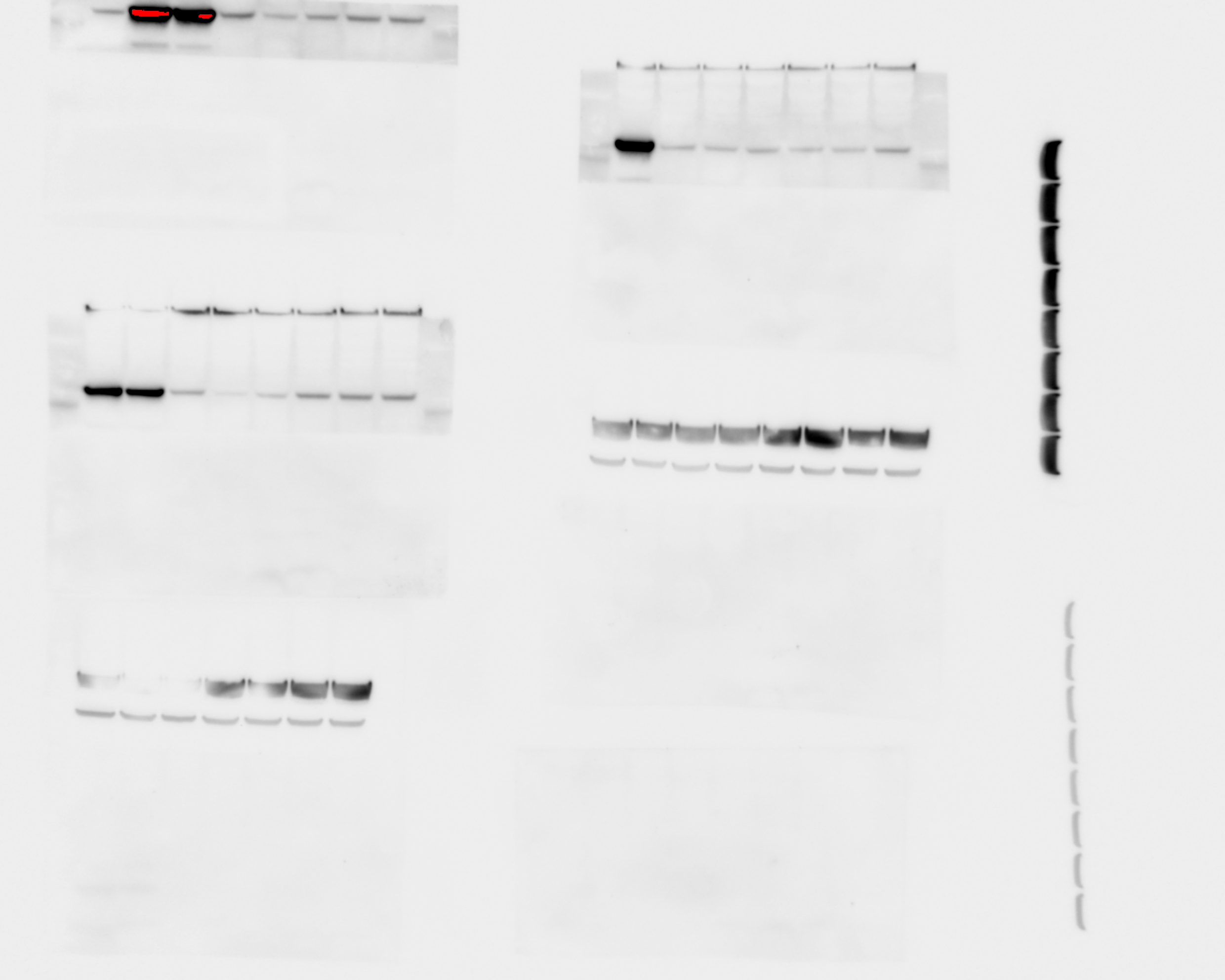

Supplement: Supplementary file 5 — Source data Fig. 5 [file 44319_2024_244_MOESM5_ESM.zip › Figure 5/5F/EGFP.tif]

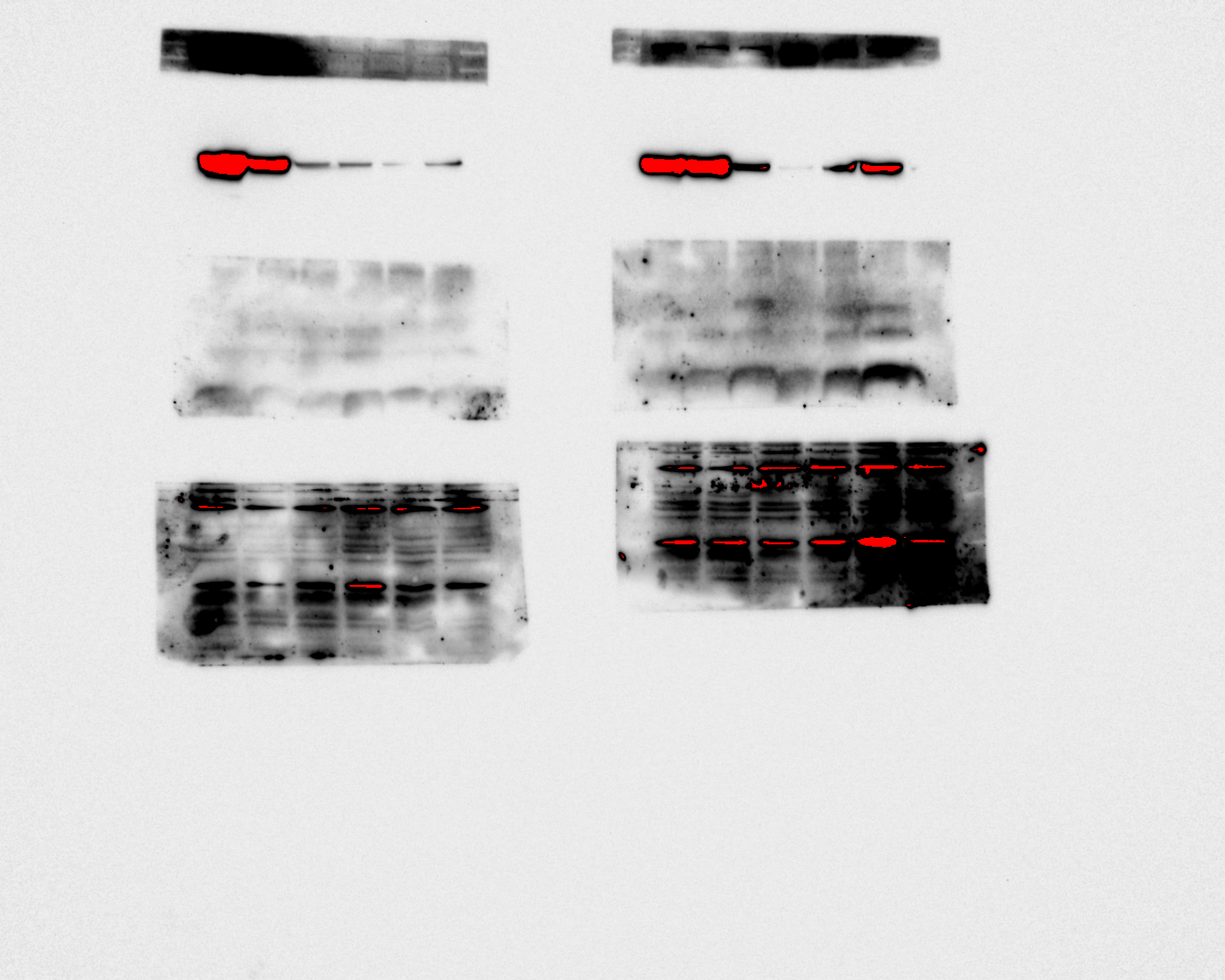

Supplement: Supplementary file 5 — Source data Fig. 5 [file 44319_2024_244_MOESM5_ESM.zip › Figure 5/5F/NPY.tif]

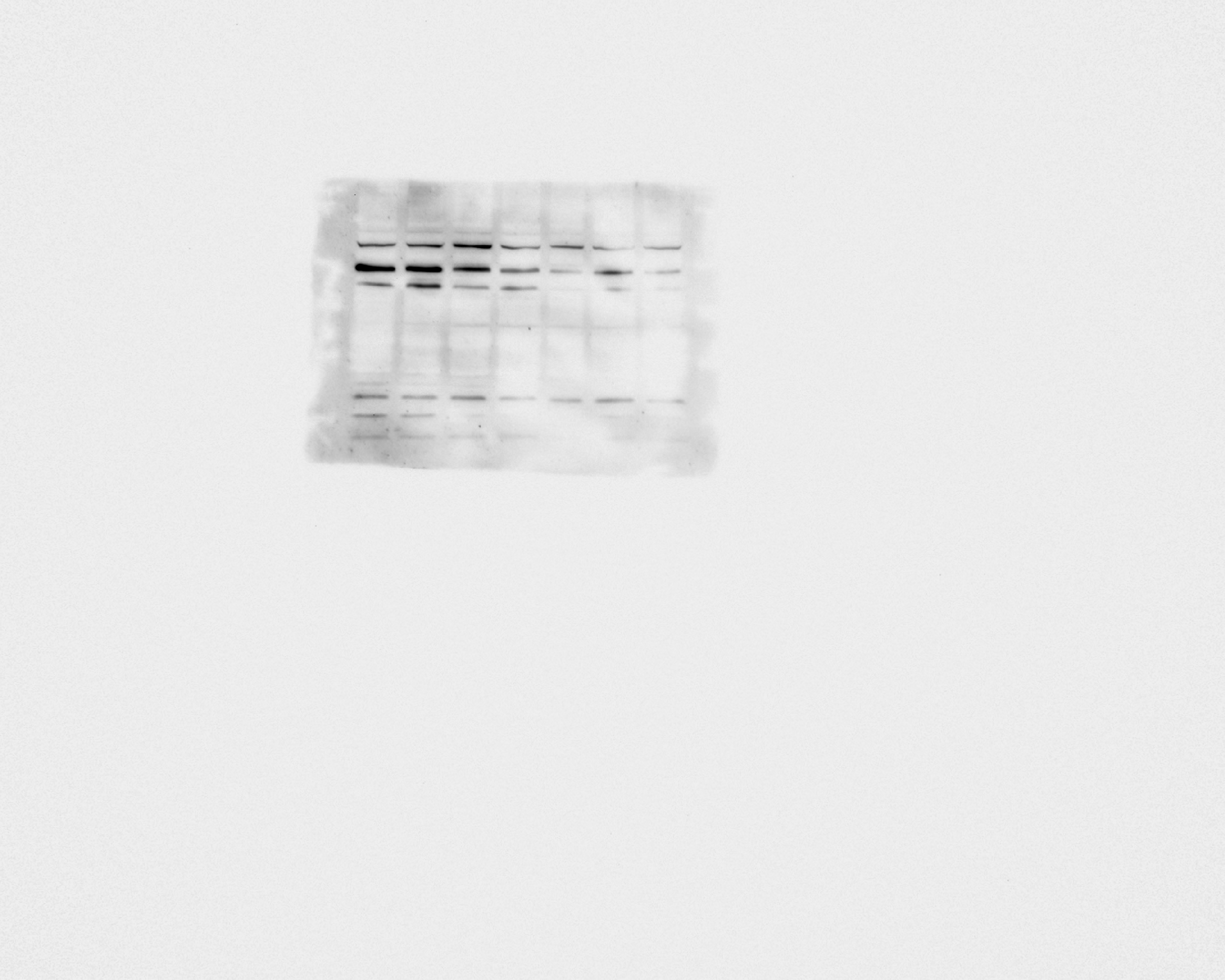

Supplement: Supplementary file 5 — Source data Fig. 5 [file 44319_2024_244_MOESM5_ESM.zip › Figure 5/5H/Calnexin.tif]

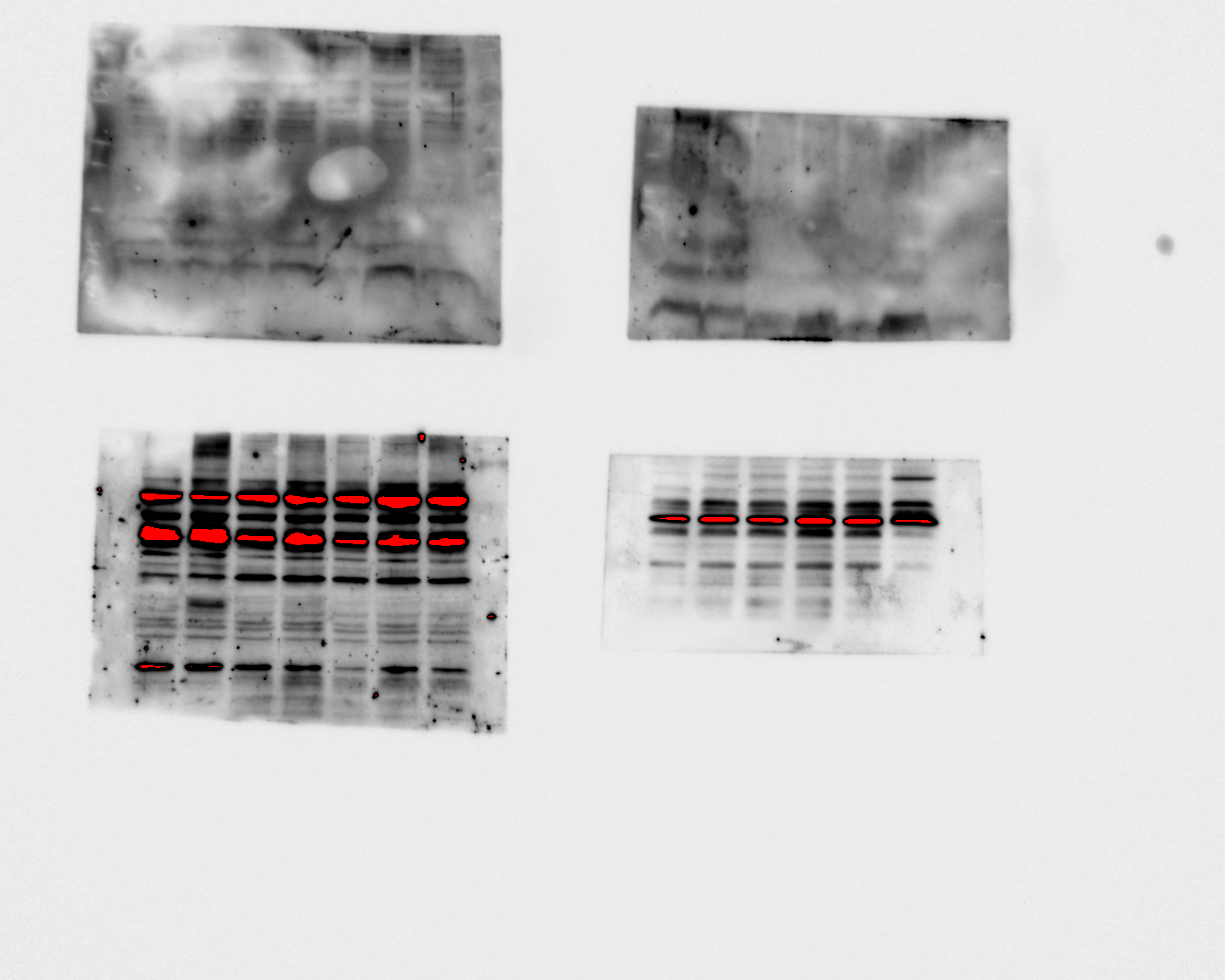

Supplement: Supplementary file 5 — Source data Fig. 5 [file 44319_2024_244_MOESM5_ESM.zip › Figure 5/5H/Y2.tif]

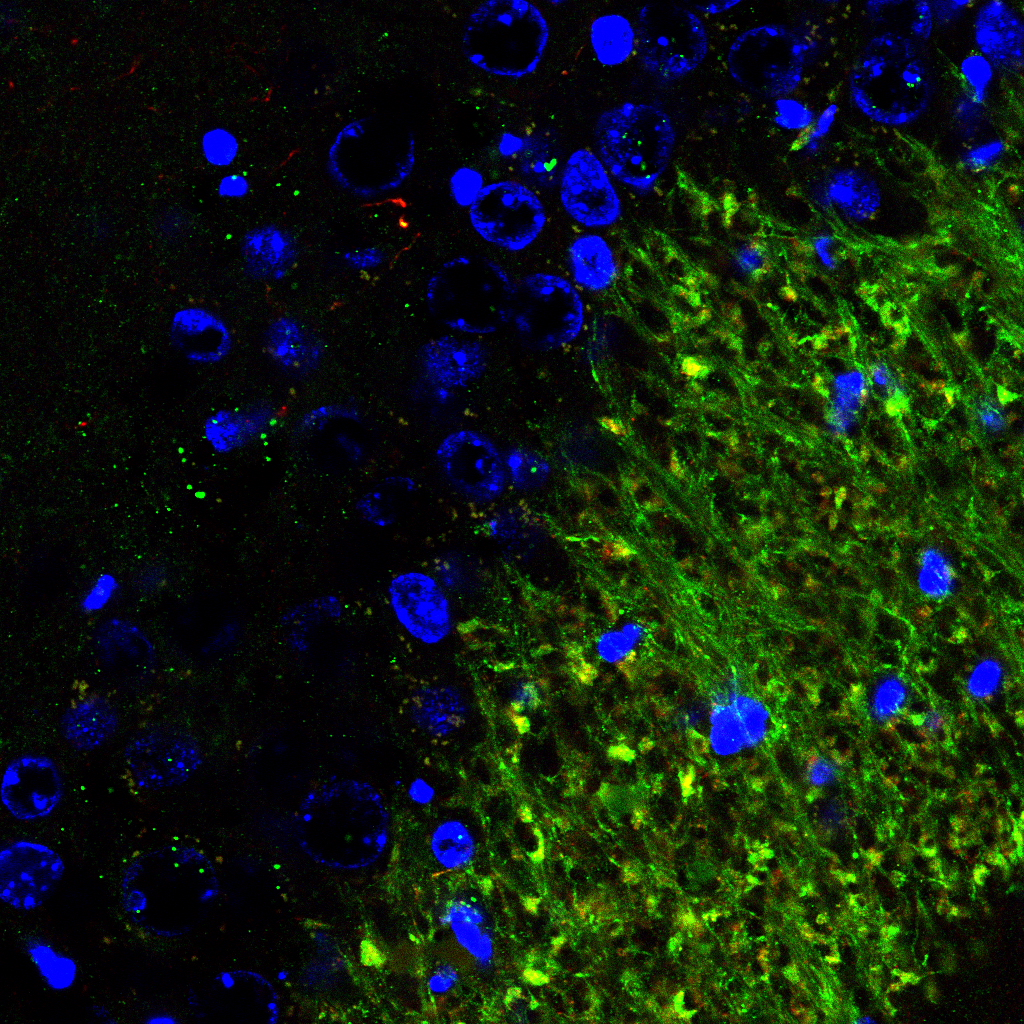

Supplement: Supplementary file 5 — Source data Fig. 5 [file 44319_2024_244_MOESM5_ESM.zip › Figure 5/5C/5C-5'''.tif]

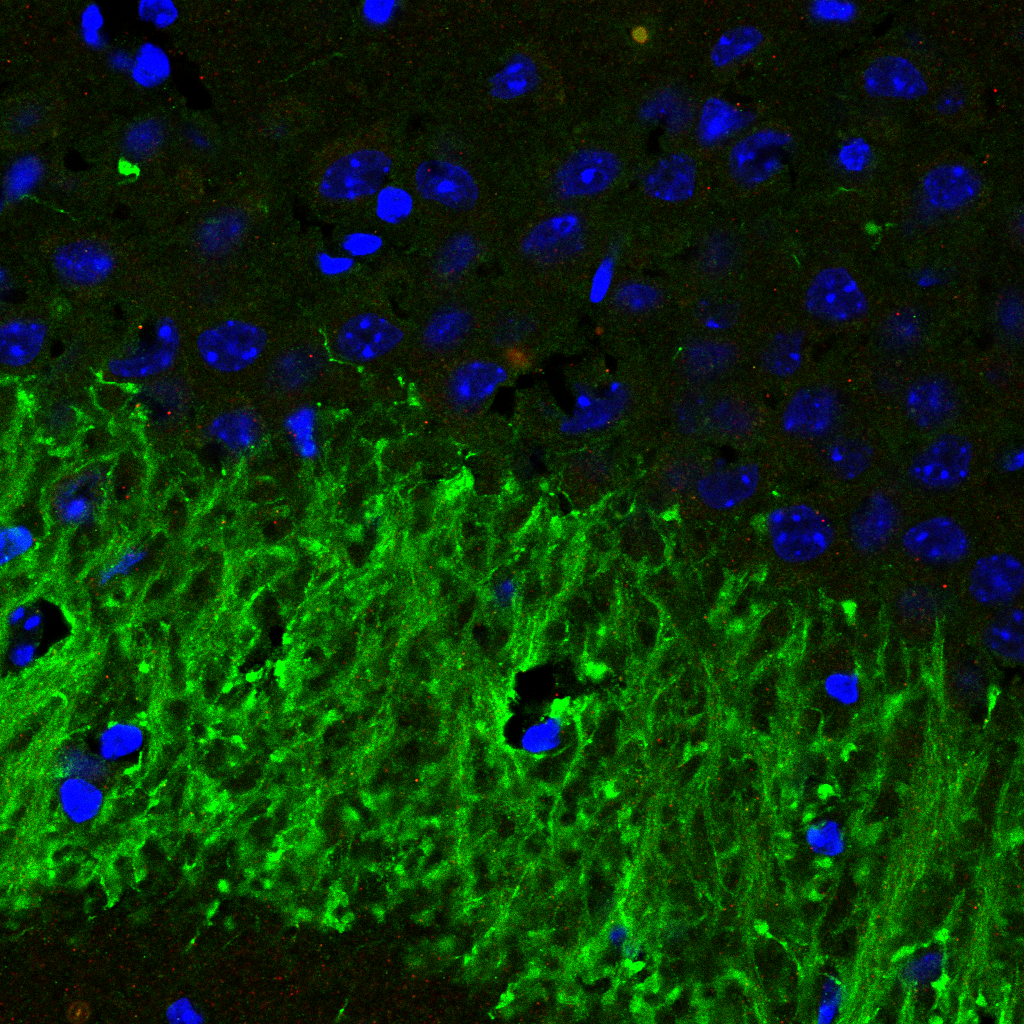

Supplement: Supplementary file 5 — Source data Fig. 5 [file 44319_2024_244_MOESM5_ESM.zip › Figure 5/5D/5D-5'''.tif]

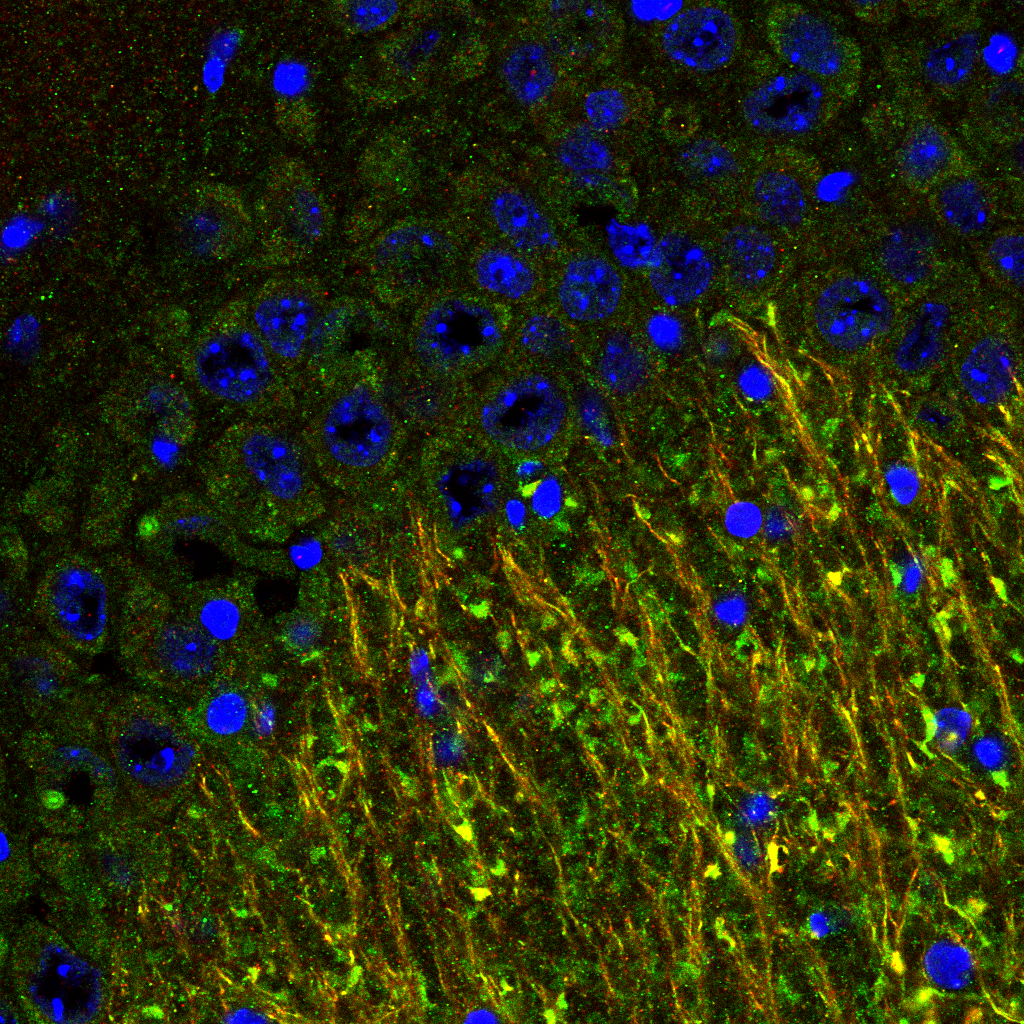

Supplement: Supplementary file 5 — Source data Fig. 5 [file 44319_2024_244_MOESM5_ESM.zip › Figure 5/5E/5E-5'''.tif]

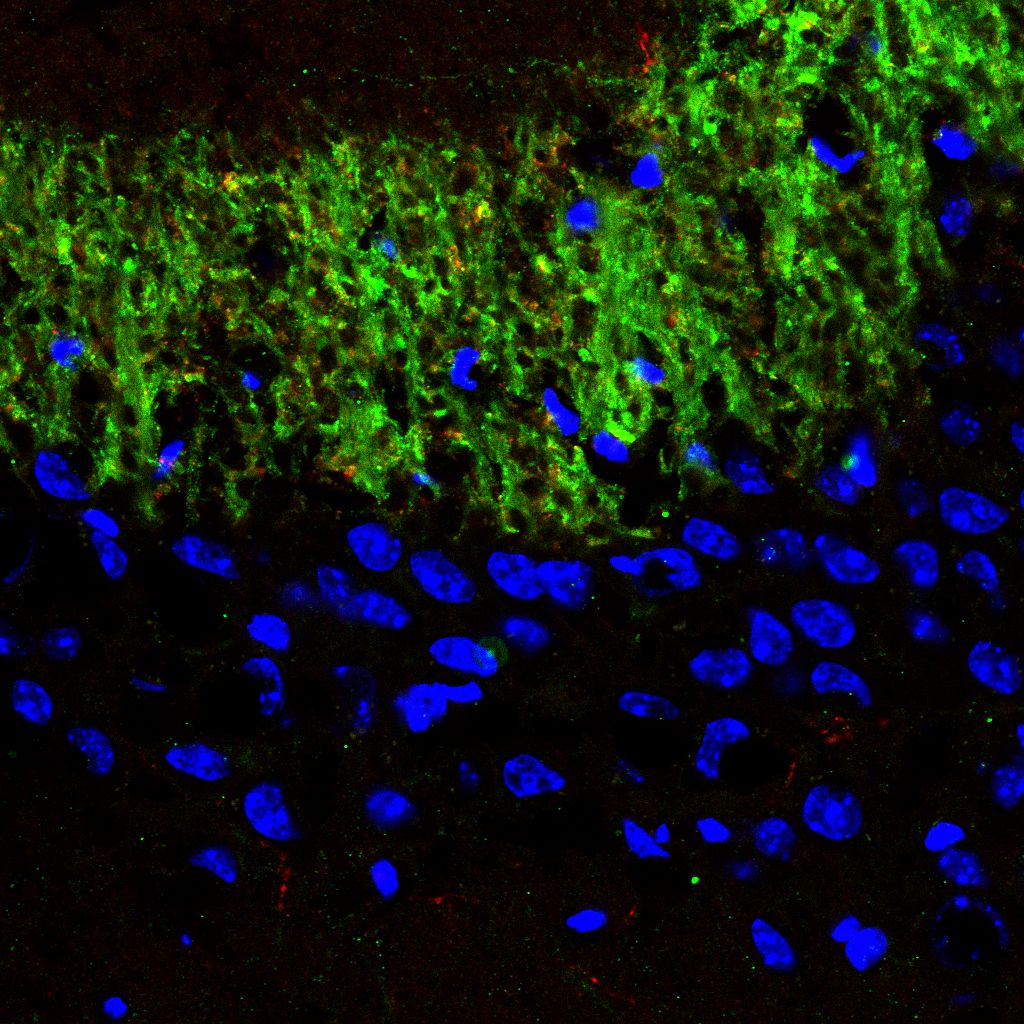

Supplement: Supplementary file 5 — Source data Fig. 5 [file 44319_2024_244_MOESM5_ESM.zip › Figure 5/5B/5B-5'''.tif]

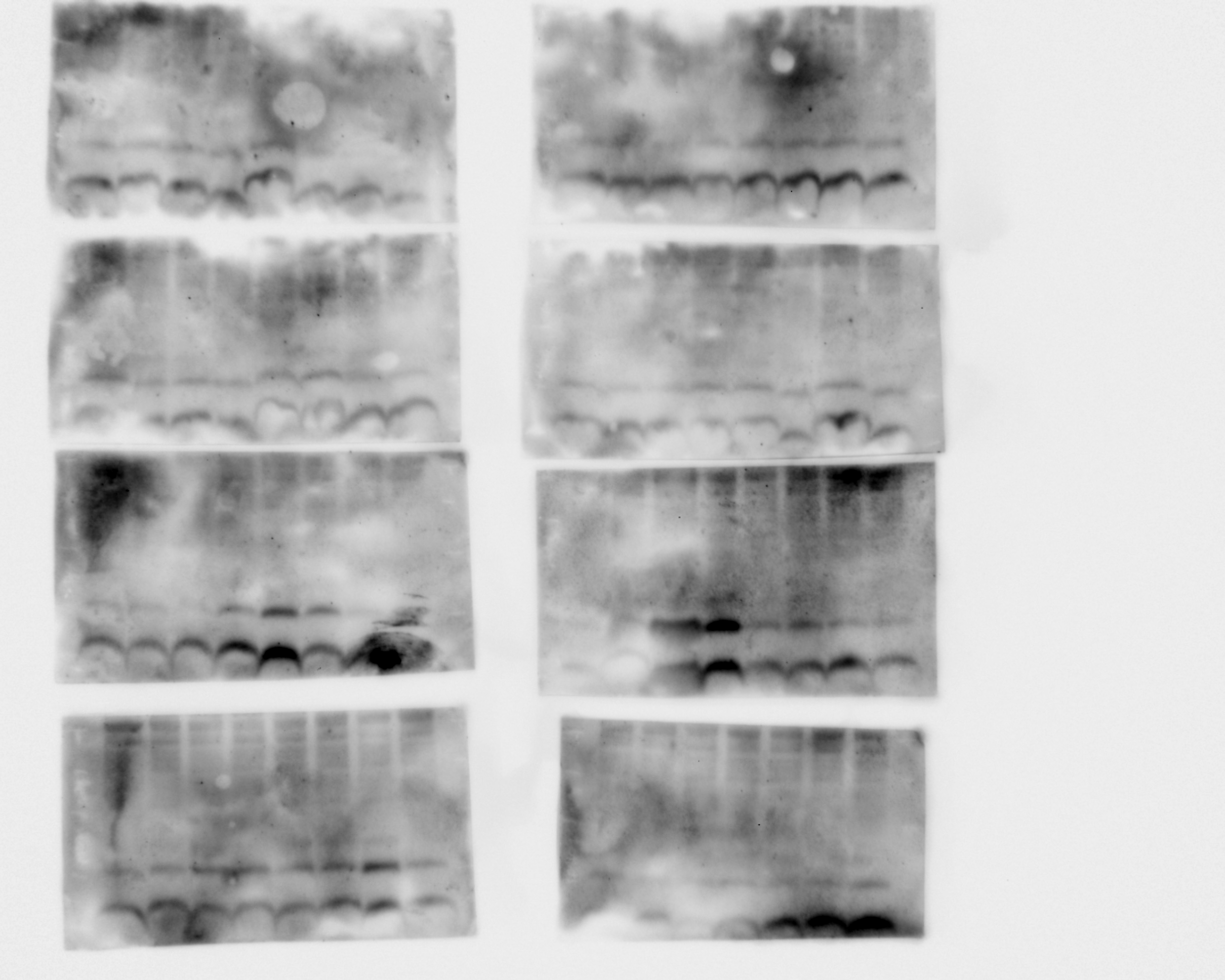

Supplement: Supplementary file 6 — Source data Fig. 6 [file 44319_2024_244_MOESM6_ESM.zip › Figure 6/6A/NPY.tif]

## Slide 1
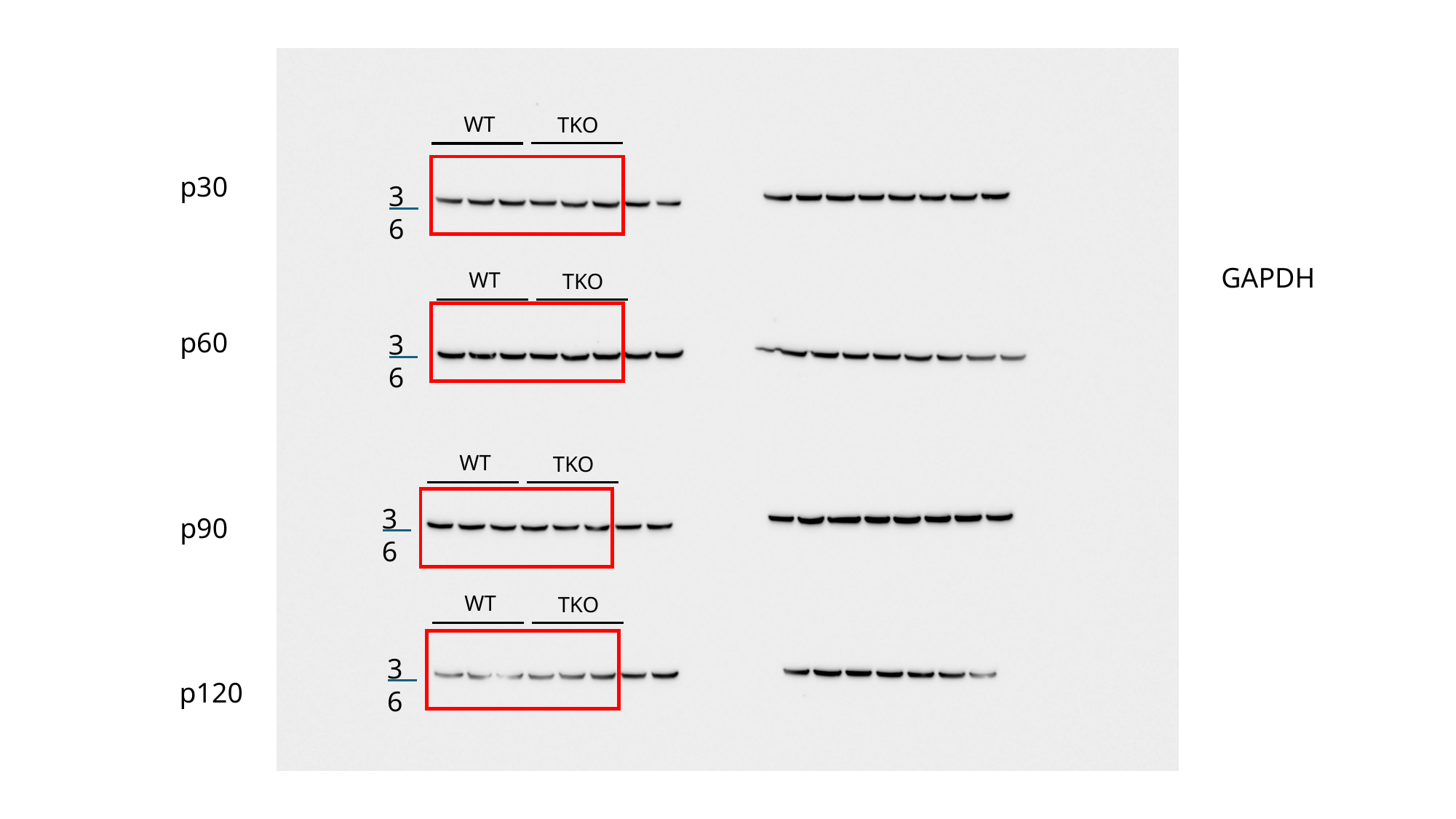

WT
TKO
p30
36
GAPDH
WT
TKO
p60
36
WT
TKO
36
p90
WT
TKO
36
p120

## Slide 2
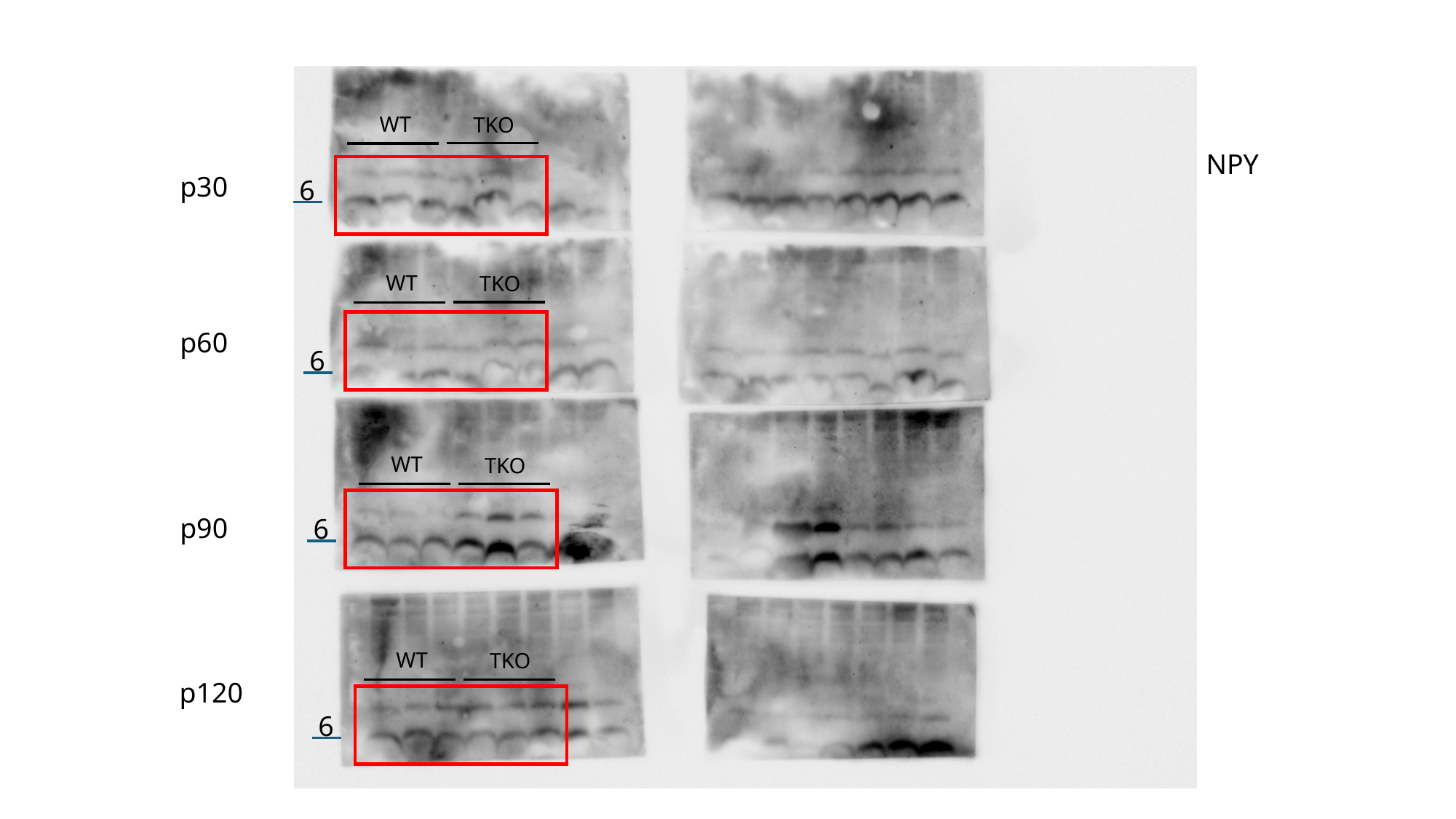

WT
TKO
NPY
p30
6
WT
TKO
p60
6
WT
TKO
p90
6
WT
TKO
p120
6

Supplement: Supplementary file 6 — Source data Fig. 6 [file 44319_2024_244_MOESM6_ESM.zip › Figure 6/6A/Figure 6.pptx]

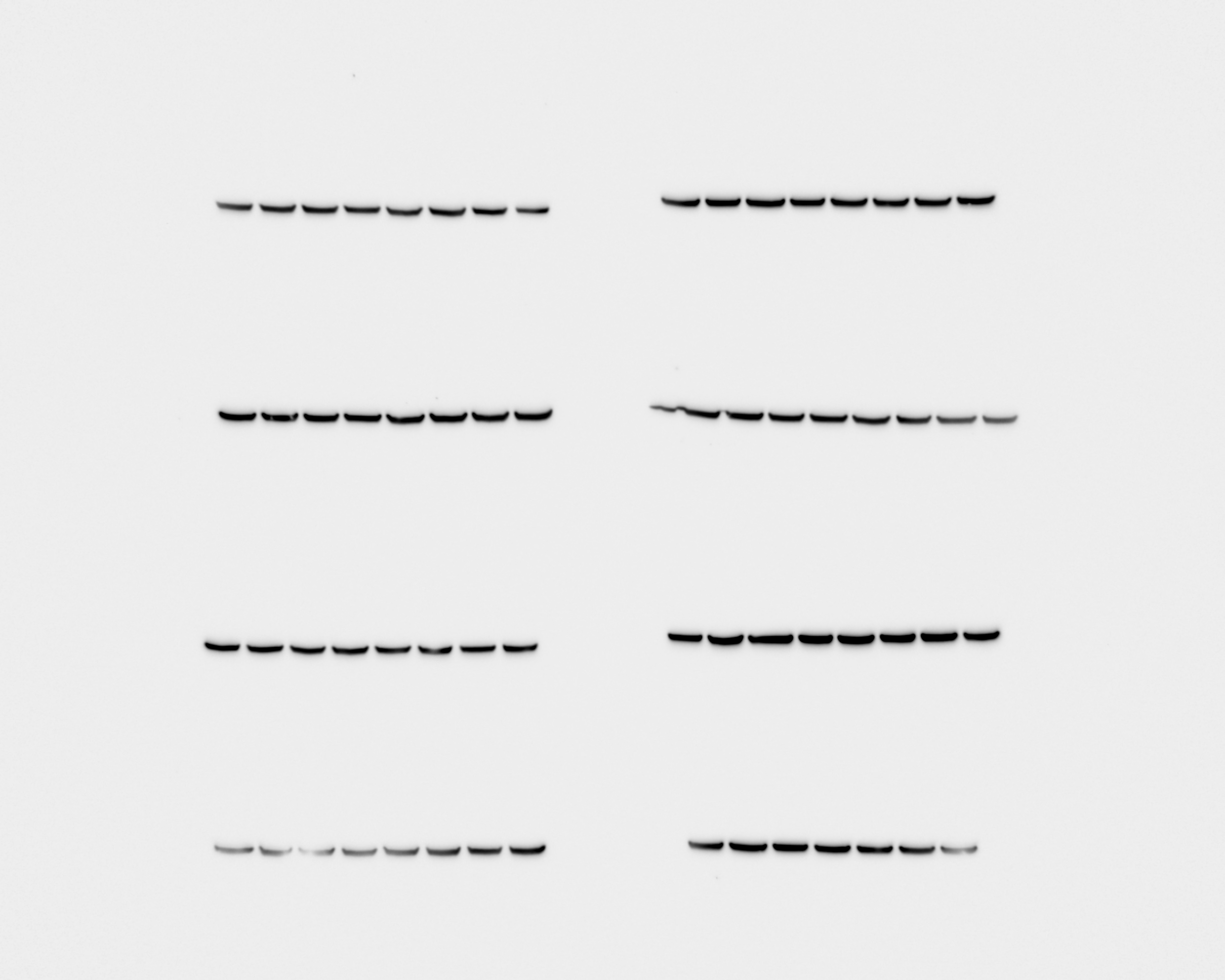

Supplement: Supplementary file 6 — Source data Fig. 6 [file 44319_2024_244_MOESM6_ESM.zip › Figure 6/6A/GAPDH.tif]

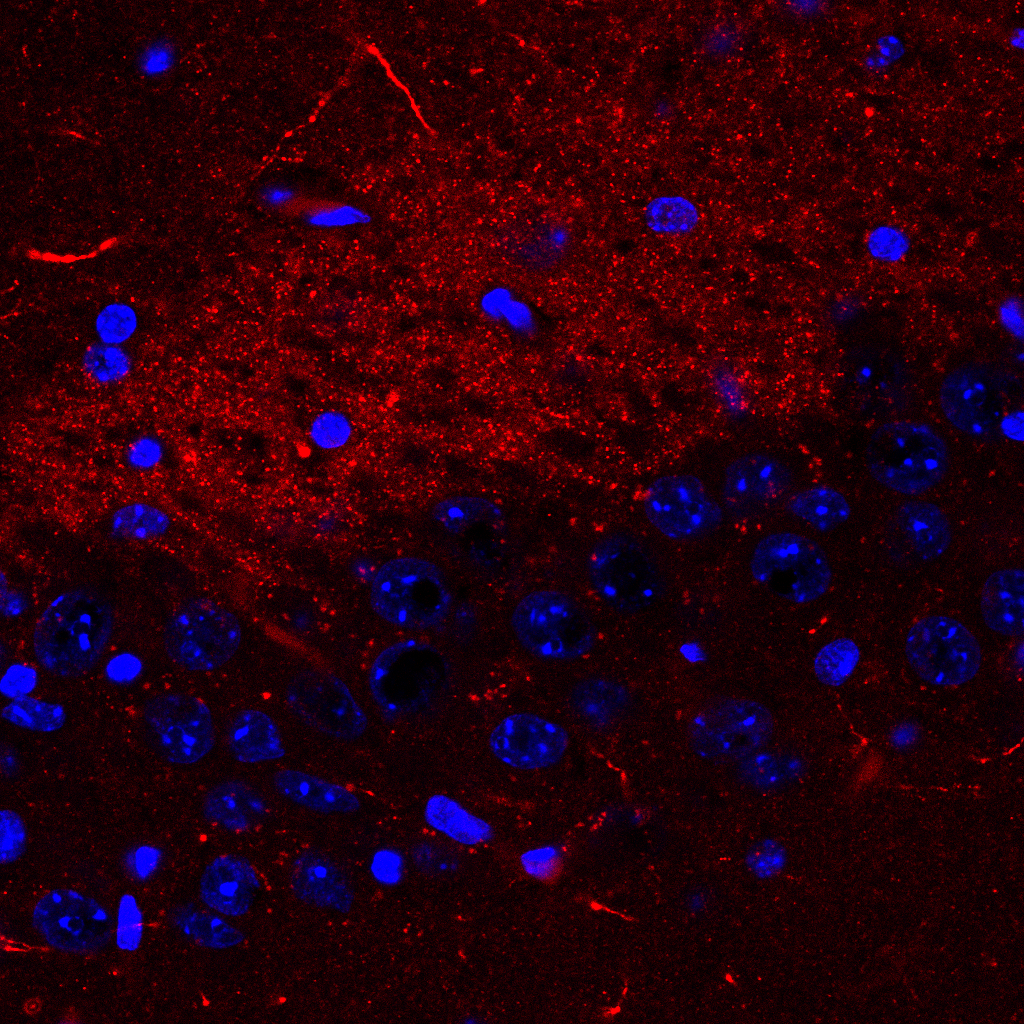

Supplement: Supplementary file 6 — Source data Fig. 6 [file 44319_2024_244_MOESM6_ESM.zip › Figure 6/6D/6D-D'''.tif]

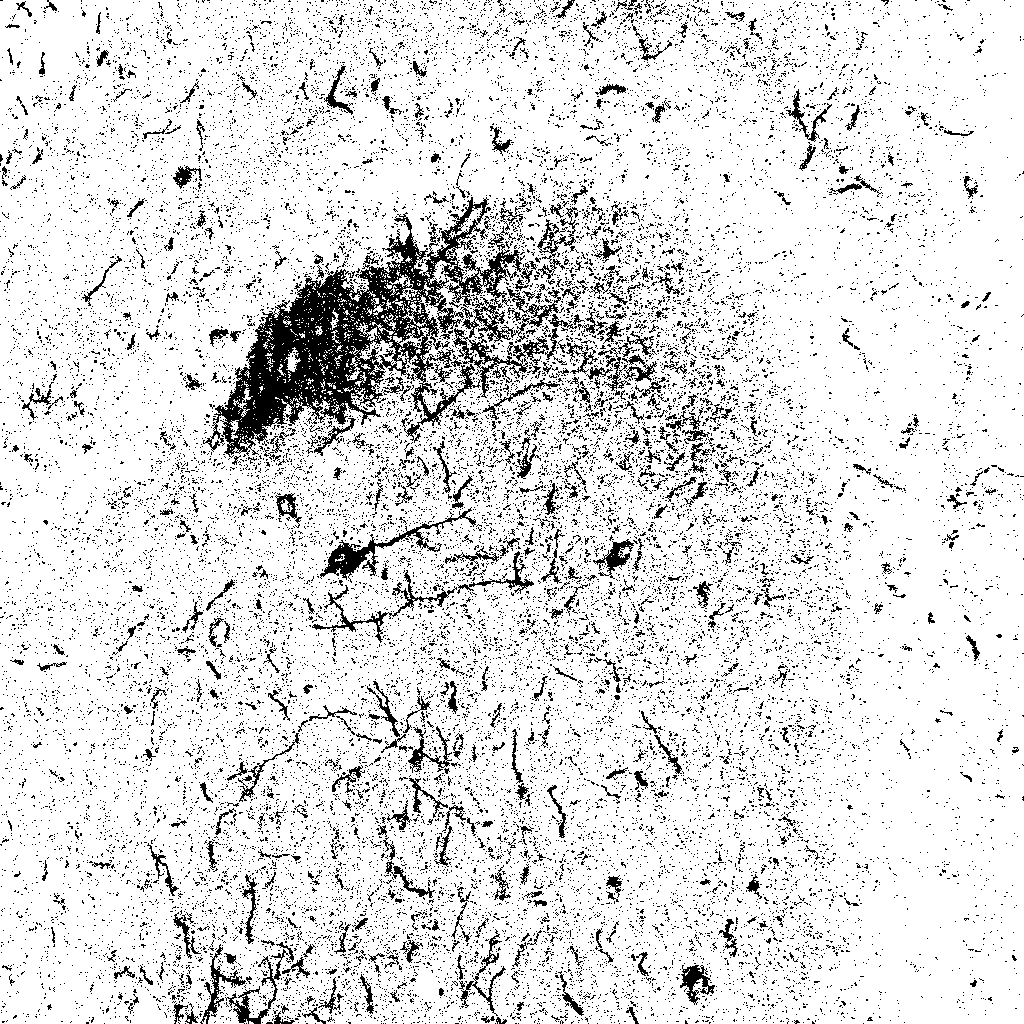

Supplement: Supplementary file 6 — Source data Fig. 6 [file 44319_2024_244_MOESM6_ESM.zip › Figure 6/6D/6D-D''.tif]

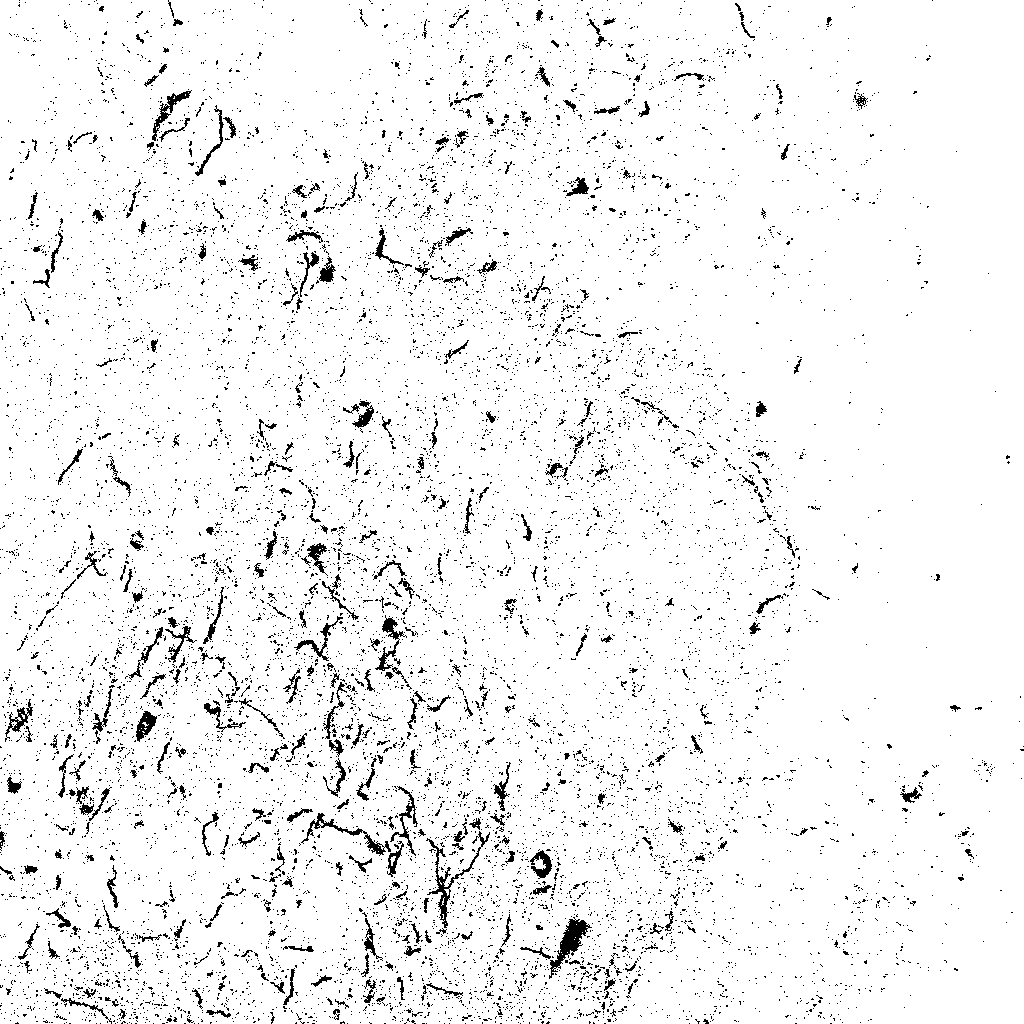

Supplement: Supplementary file 6 — Source data Fig. 6 [file 44319_2024_244_MOESM6_ESM.zip › Figure 6/6C/6C-C''.tif]

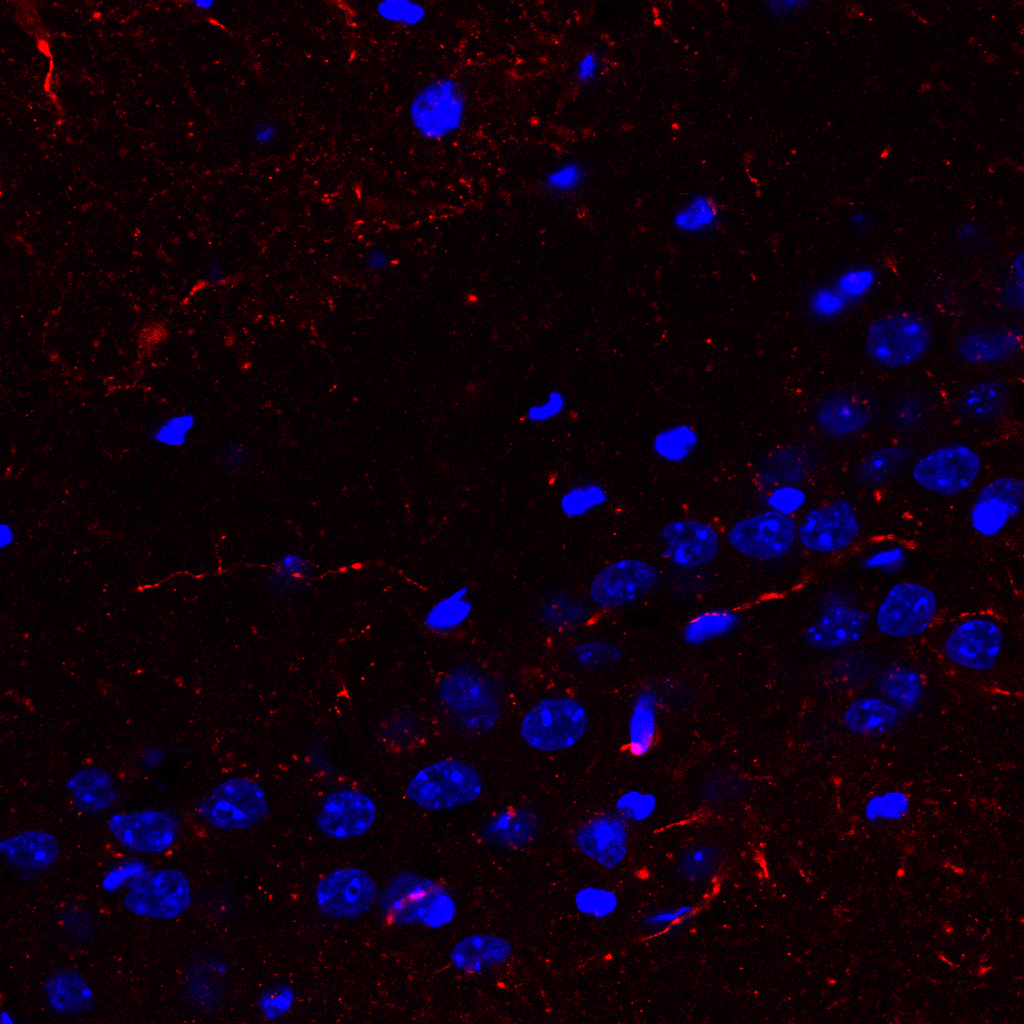

Supplement: Supplementary file 6 — Source data Fig. 6 [file 44319_2024_244_MOESM6_ESM.zip › Figure 6/6C/6C-C'''.tif]

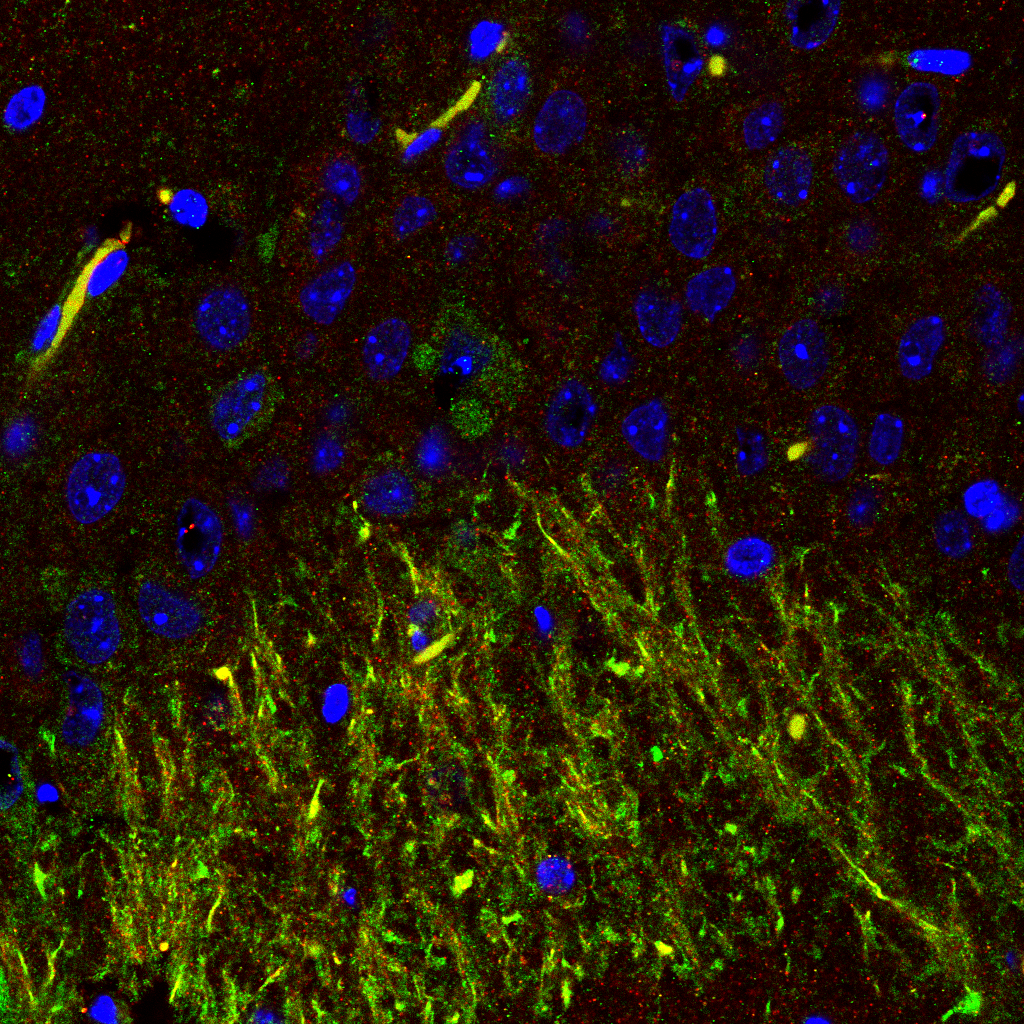

Supplement: Supplementary file 7 — Source data Fig. 7 [file 44319_2024_244_MOESM7_ESM.zip › Figure 7/7J/7J-J'''.tif]

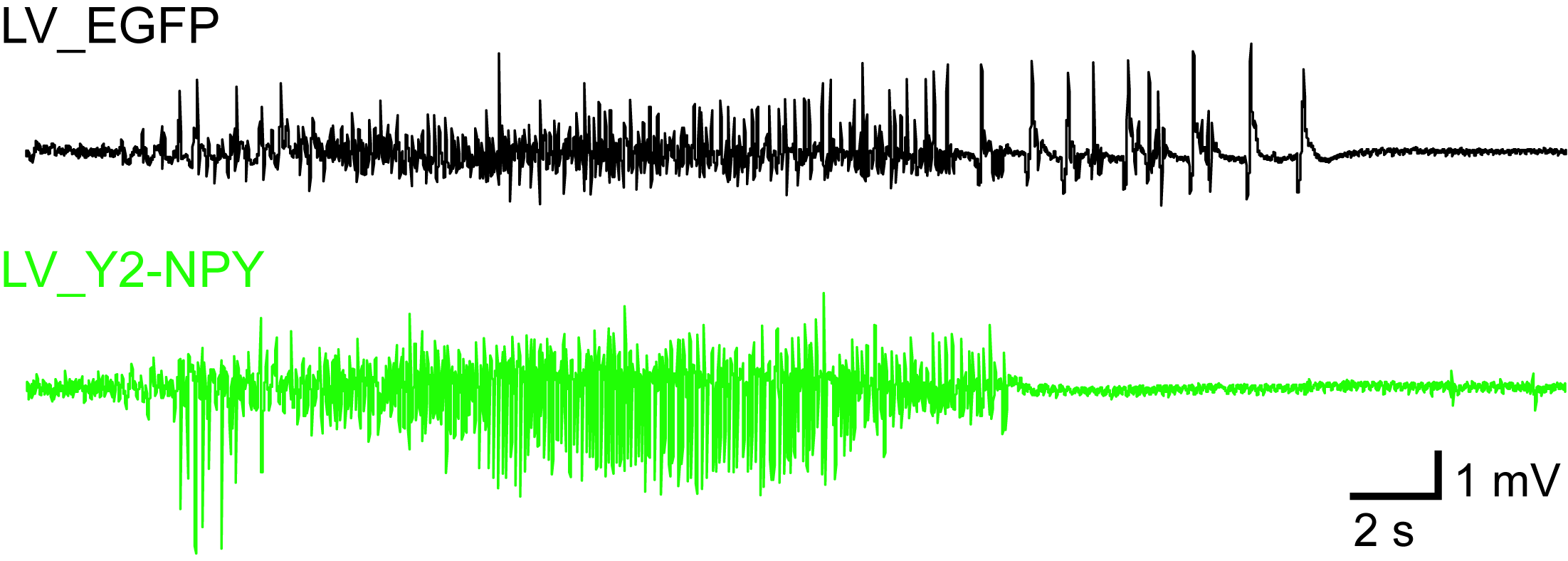

Supplement: Supplementary file 7 — Source data Fig. 7 [file 44319_2024_244_MOESM7_ESM.zip › Figure 7/7B/Figure 7B.tif]

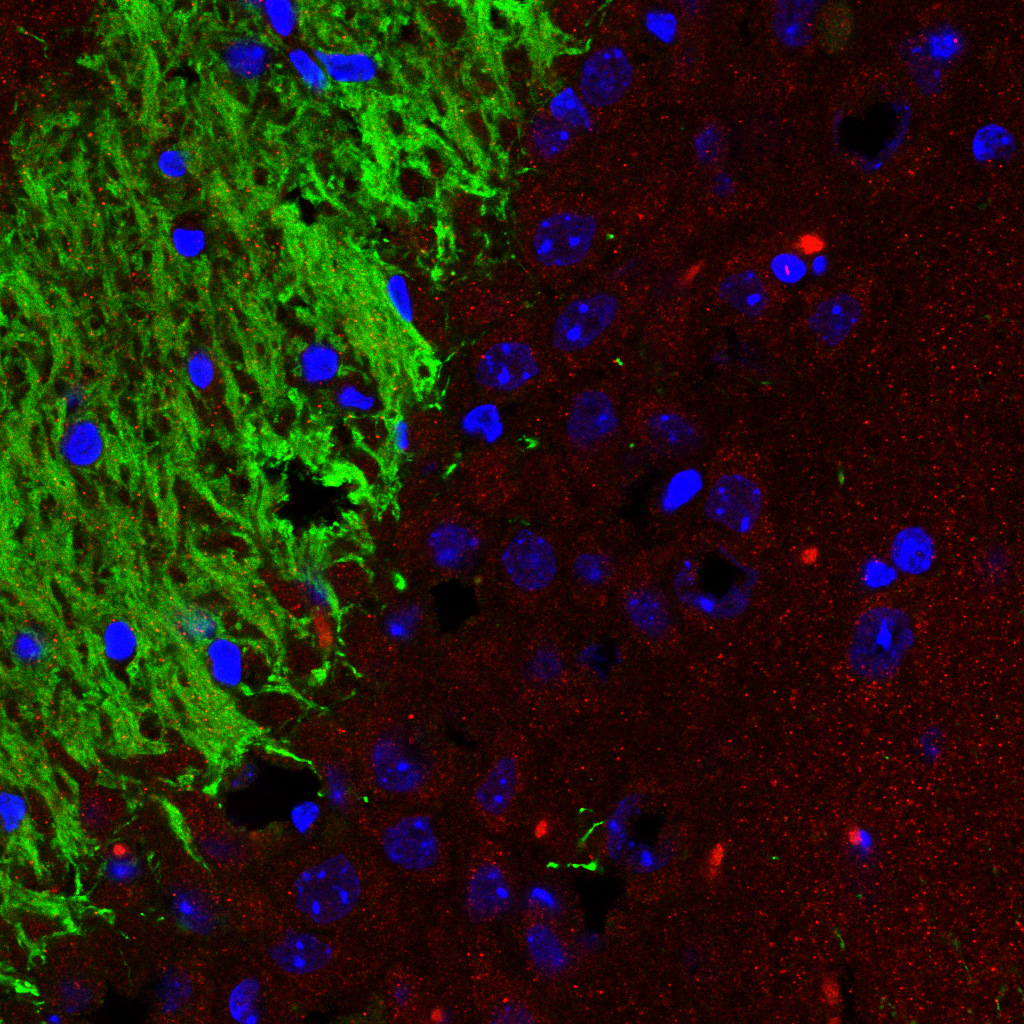

Supplement: Supplementary file 7 — Source data Fig. 7 [file 44319_2024_244_MOESM7_ESM.zip › Figure 7/7I/7I-I'''.tif]

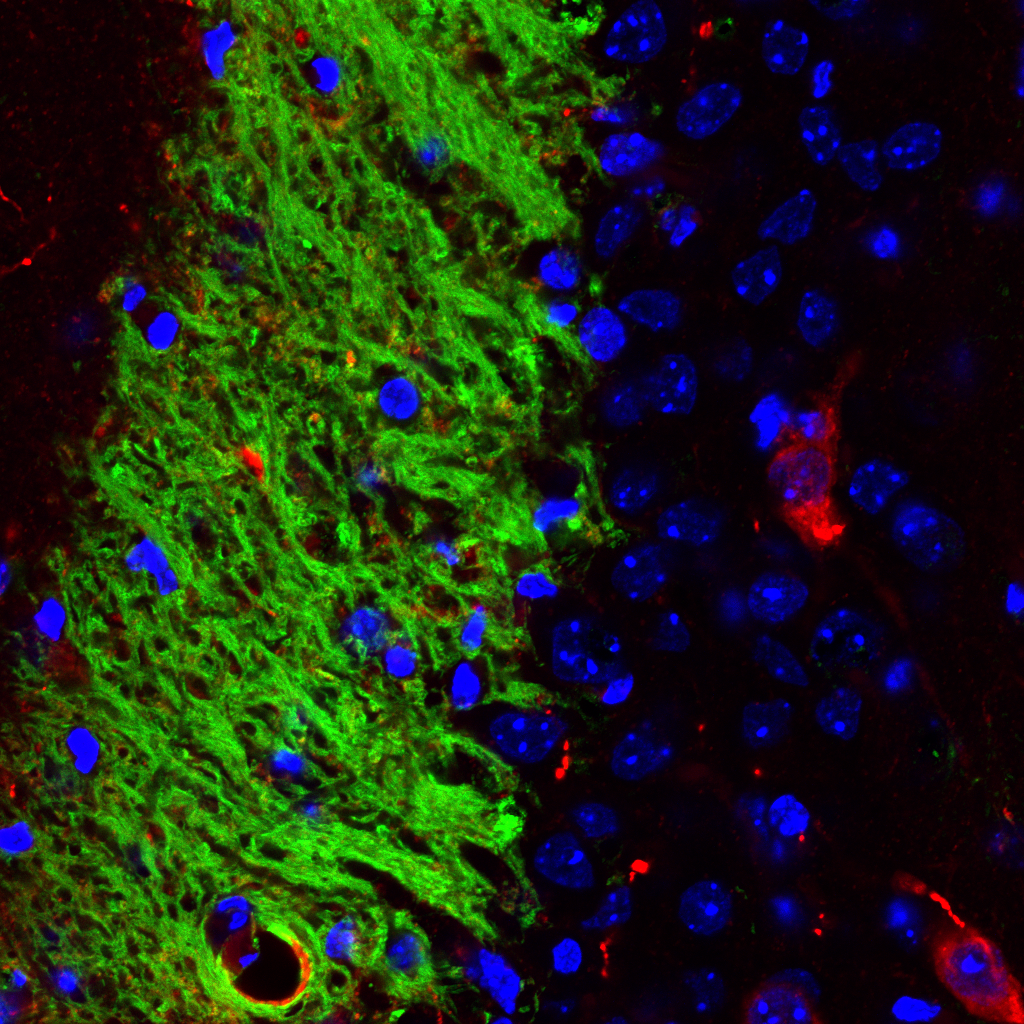

Supplement: Supplementary file 7 — Source data Fig. 7 [file 44319_2024_244_MOESM7_ESM.zip › Figure 7/7G/7G-G'''.tif]

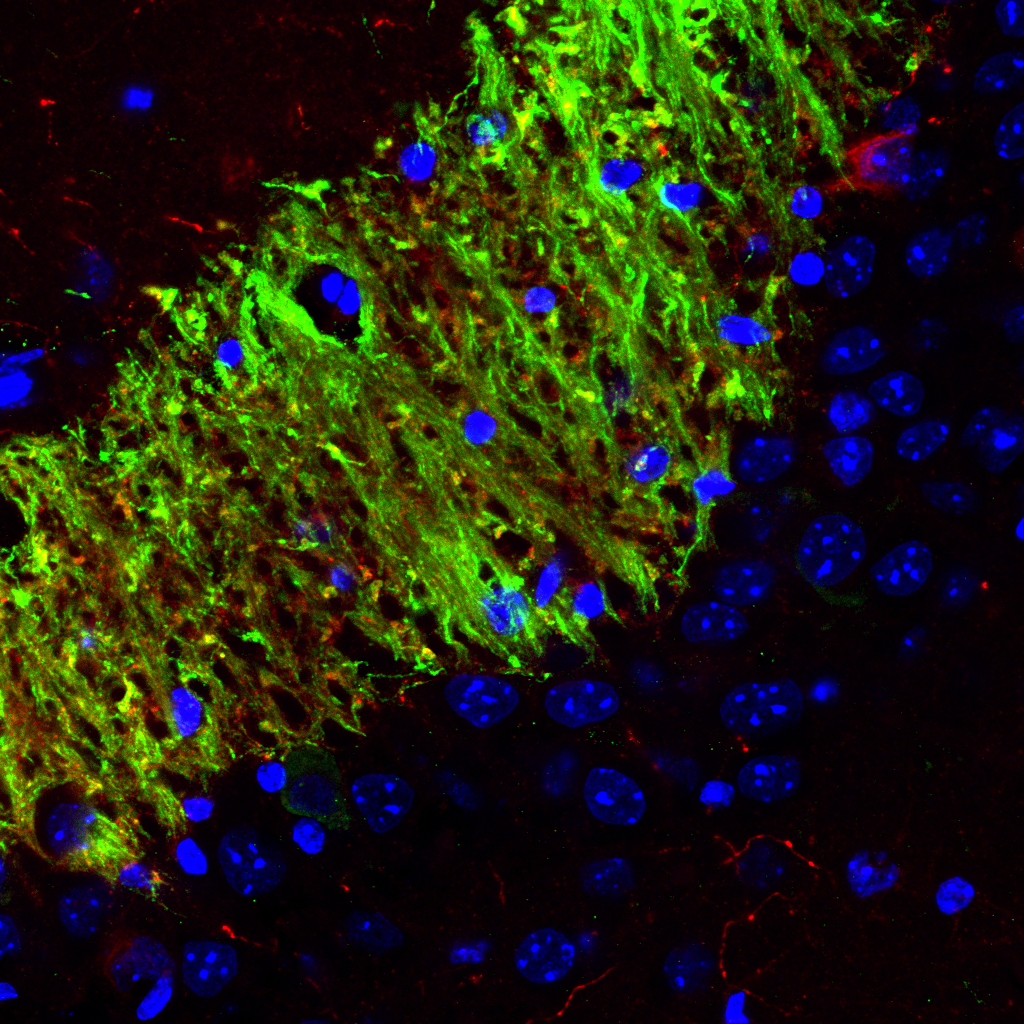

Supplement: Supplementary file 7 — Source data Fig. 7 [file 44319_2024_244_MOESM7_ESM.zip › Figure 7/7H/7H-H'''.tif]
