# Supplementary material for: Gene therapy for epilepsy targeting neuropeptide Y and its Y2 receptor to dentate gyrus granule cells
Source: EMBO Rep. 2024 Sep 9;25(10):20. doi: 10.1038/s44319-024-00244-0 (PMC11467199; doi:10.1038/s44319-024-00244-0)
Supplement: Supplementary file 5 — Source data Fig. 5 [file 44319_2024_244_MOESM5_ESM.zip › Figure 5/5F/Figure 5F.pptx]

## Slide 1
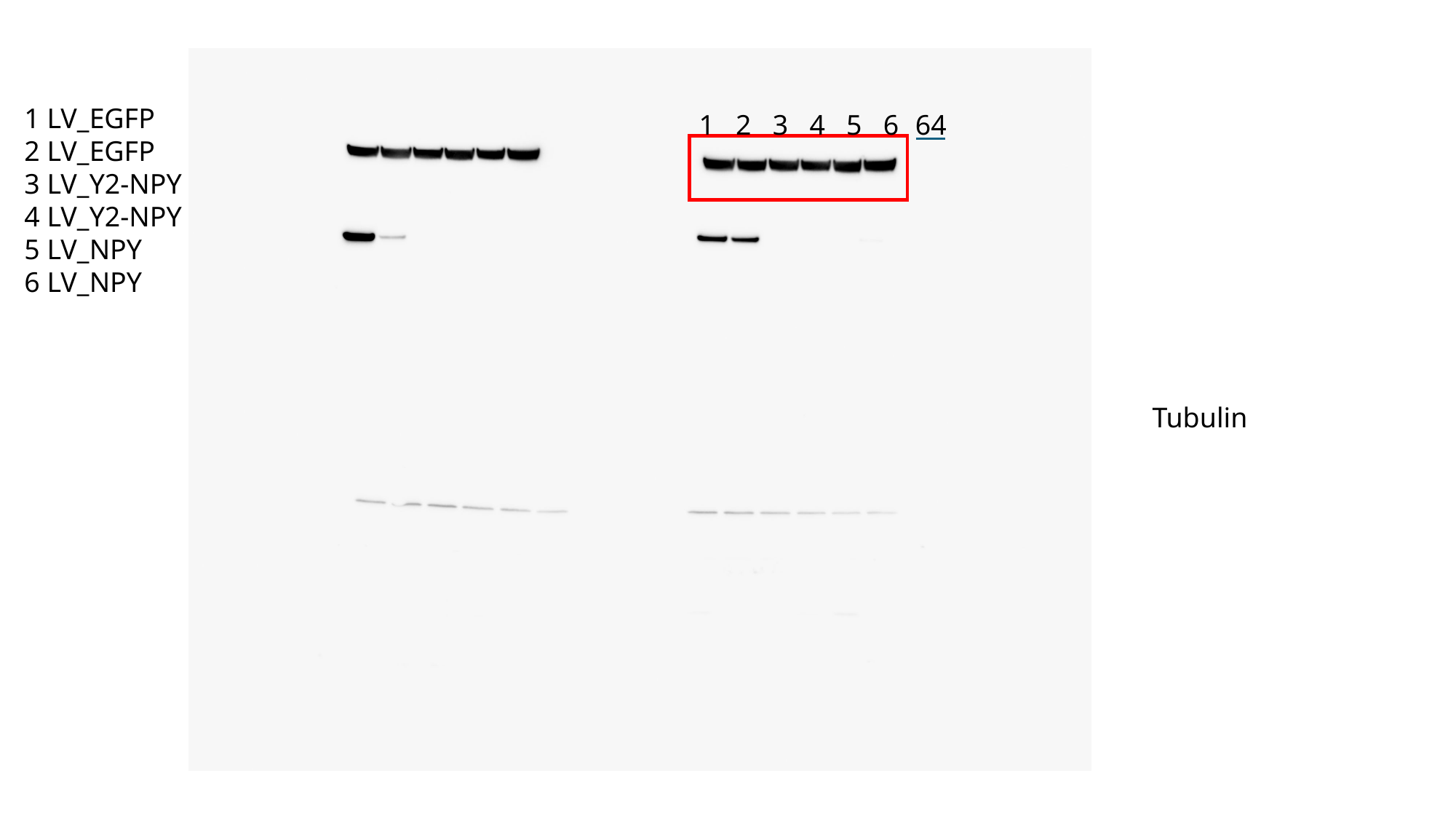

1 LV_EGFP
2 LV_EGFP
3 LV_Y2-NPY
4 LV_Y2-NPY
5 LV_NPY
6 LV_NPY
1 2 3 4 5 6
64
Tubulin

## Slide 2
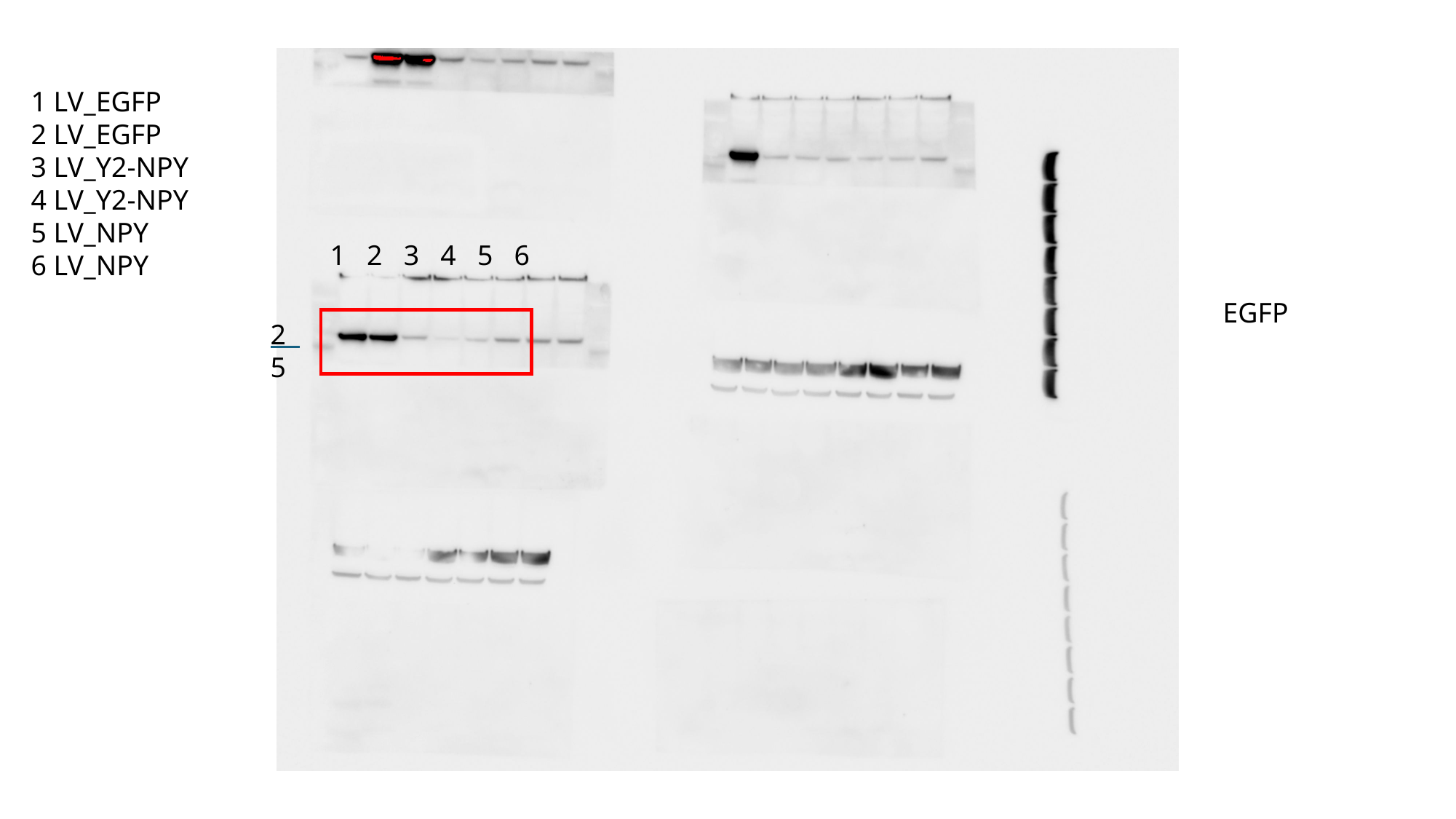

64
1 LV_EGFP
2 LV_EGFP
3 LV_Y2-NPY
4 LV_Y2-NPY
5 LV_NPY
6 LV_NPY
Tubulin
1 2 3 4 5 6
15
EGFP
NPY
25
10
5
EGFP
Y2-Flag

## Slide 3
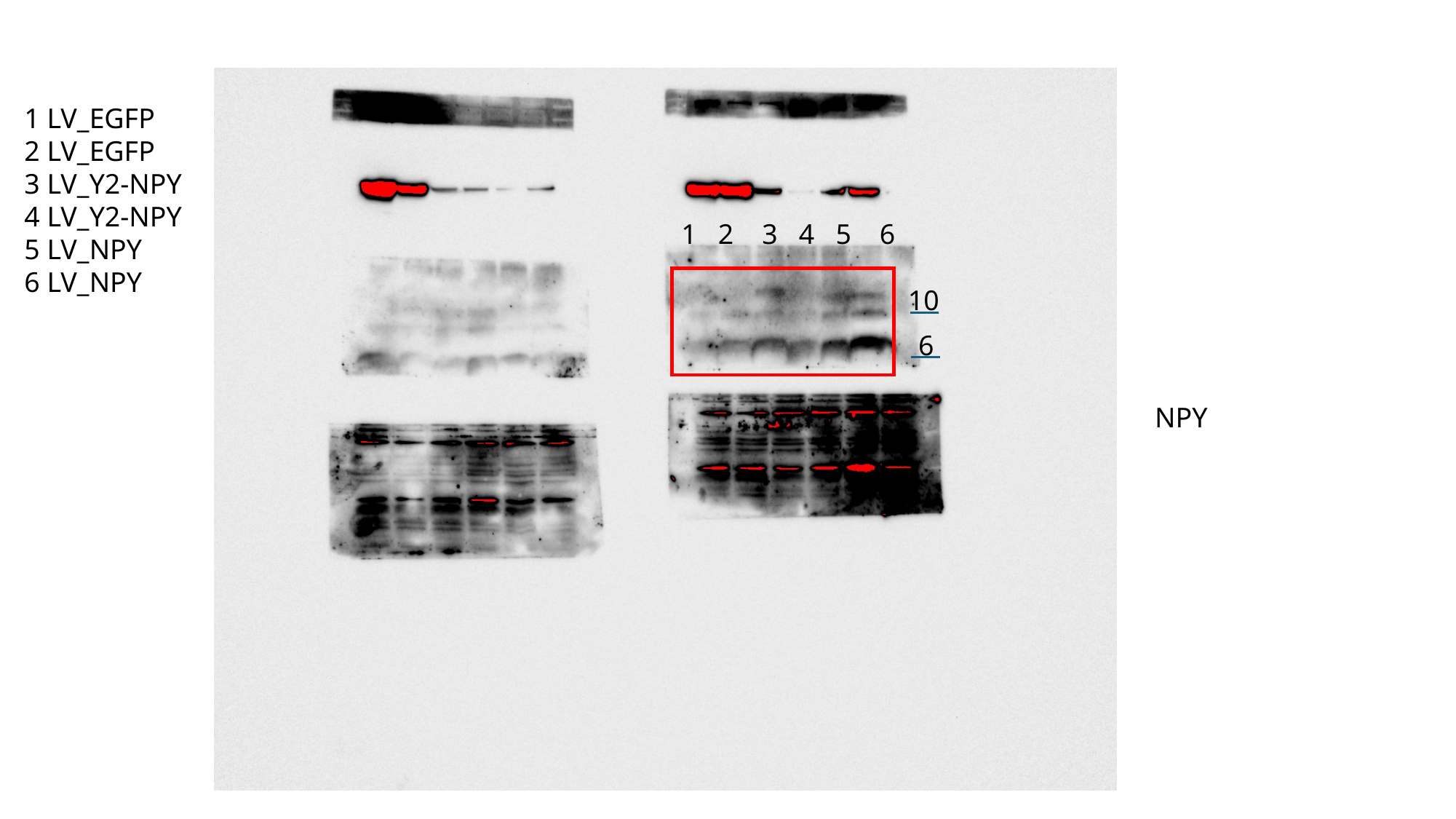

1 LV_EGFP
2 LV_EGFP
3 LV_Y2-NPY
4 LV_Y2-NPY
5 LV_NPY
6 LV_NPY
1 2 3 4 5 6
10
6
NPY
