# Supplementary material for: Gene therapy for epilepsy targeting neuropeptide Y and its Y2 receptor to dentate gyrus granule cells
Source: EMBO Rep. 2024 Sep 9;25(10):20. doi: 10.1038/s44319-024-00244-0 (PMC11467199; doi:10.1038/s44319-024-00244-0)
Supplement: Supplementary file 5 — Source data Fig. 5 [file 44319_2024_244_MOESM5_ESM.zip › Figure 5/5H/Figure 5H.pptx]

## Slide 1
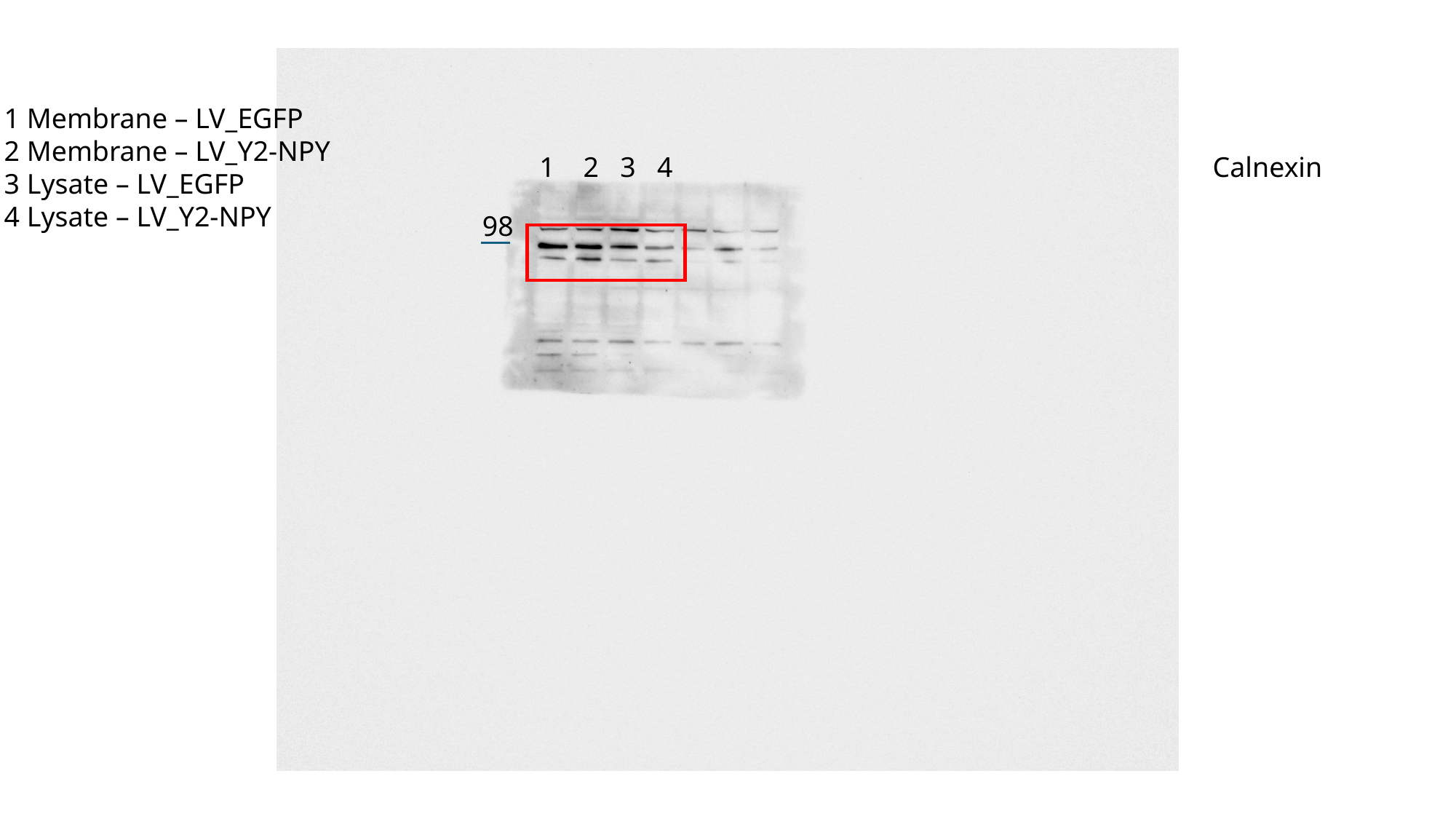

1 Membrane – LV_EGFP
2 Membrane – LV_Y2-NPY
3 Lysate – LV_EGFP
4 Lysate – LV_Y2-NPY
1 2 3 4
Calnexin
98

## Slide 2
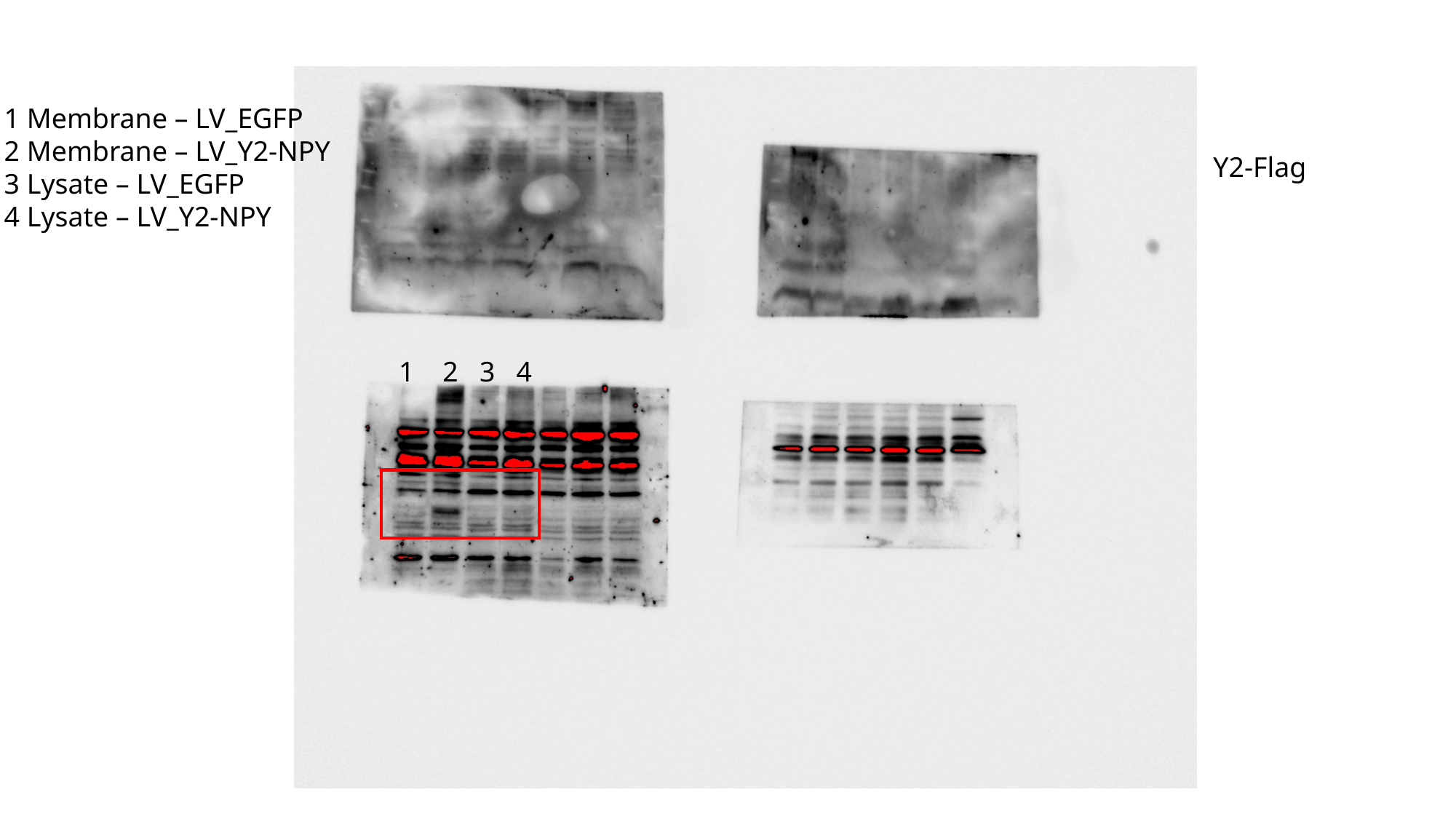

1 Membrane – LV_EGFP
2 Membrane – LV_Y2-NPY
3 Lysate – LV_EGFP
4 Lysate – LV_Y2-NPY
Y2-Flag
1 2 3 4
