# Supplementary material for: Gene therapy for epilepsy targeting neuropeptide Y and its Y2 receptor to dentate gyrus granule cells
Source: EMBO Rep. 2024 Sep 9;25(10):20. doi: 10.1038/s44319-024-00244-0 (PMC11467199; doi:10.1038/s44319-024-00244-0)
Supplement: Supplementary file 9 — Expanded View Figures [file 44319_2024_244_MOESM9_ESM.pdf]

Expanded View Figures

Figure EV1. Electrophysiological characterization of LV transduced primary rat hippocampal neurons.

(**A**–**C**) Voltage response of LV transduced neurons to suprathreshold depolarizing currents steps. (**D**–**H**) Dot plots summarizing average resting membrane potential, action potential threshold, input resistance, spontaneous excitatory post-synaptic currents (sEPSCs) amplitude, and sEPSCs frequency of transduced neurons. Data were shown as mean ± SEM of 3 independent experiments (biological replicates). (**D**) LV_EGFP = −55.46 ± 1.77 mV; LV_NPY = −57.08 ± 2.07 mV; LV_Y2-NPY = −56.43 ± 2.07 mV. (**E**) LV_EGFP = −32.64 ± 0.45 mV; LV_NPY = −33.00 ± 0.66 mV; LV_Y2-NPY = −32.21 ± 1.4 mV. (**F**) LV_EGFP = 261.4 ± 20.94 MΩ; LV_NPY = 223.8 ± 44.47 MΩ; LV_Y2-NPY = 271.5 ± 42.14 MΩ. (**G**) LV_EGFP = 30.93 ± 4.12 pA; LV_NPY = 28.17 ± 3.25 pA; LV_Y2-NPY = 34.17 ± 8.49 pA. H, LV_EGFP = 2.12 ± 0.38 Hz; LV_NPY = 1.69 ± 0,32 Hz; LV_Y2-NPY = 2.63 ± 0.66 Hz. Source data are available online for this figure.

© The Author(s)

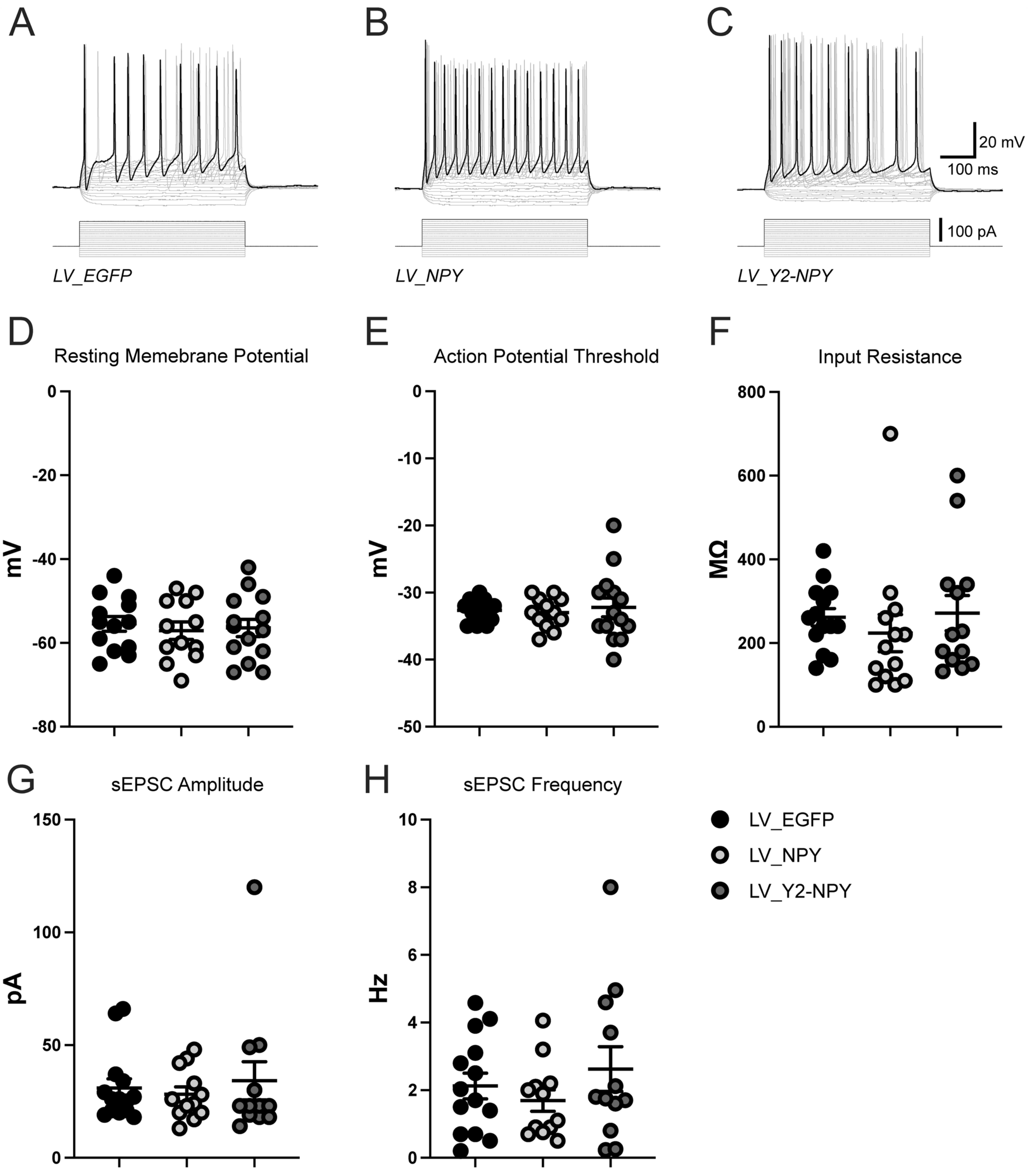

A
B
C
20 mV
100 ms
100 pA
LV_EGFP
LV_NPY
LV_Y2-NPY
D
Resting Memebrane Potential
mV
E
Action Potential Threshold
mV
F
Input Resistance
MΩ
G
sEPSC Amplitude
pA
H
sEPSC Frequency
Hz
LV_EGFP
LV_NPY
LV_Y2-NPY

 © The Author(s)

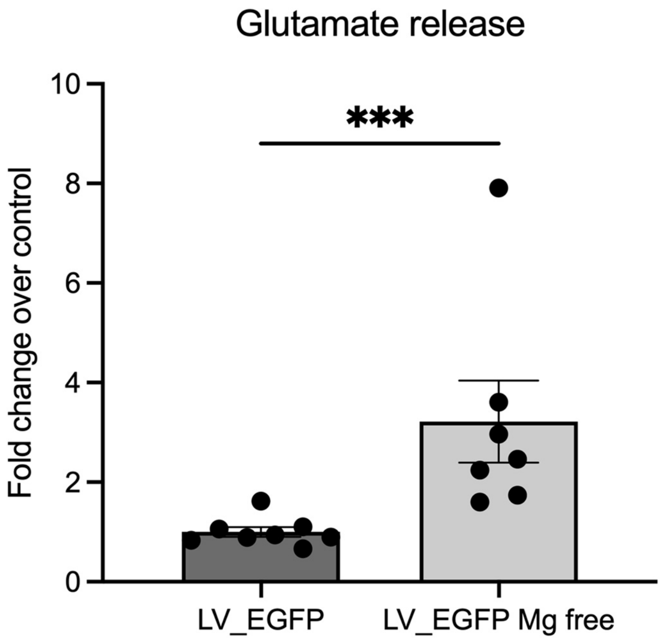

Figure EV2. Effect of Mg^{2+} removal on glutamate release.

Measurement of extracellular glutamate in transduced primary hippocampal neurons incubated for 20 min in Kreb's Ringer Hepes in the presence (LV_EGFP) or absence of Mg^{2+} (LV_EGFP Mg free). Values were normalized for total protein content and are presented as means ± SEM of at least three independent experiments (biological replicates), with glutamate levels shown as fold change over control (LV_EGFP = 1.00 ± 0.10; LV_ EGFP Mg free = 3.22 ± 0.82; $p = 0.0006$). Statistical significance was calculated using the Mann–Whitney test. ***$p < 0.0001$. Source data are available online for this figure.

© The Author(s)
